# Supplementary figures and images for: Nitrogen signaling factor triggers a respiration-like gene expression program in fission yeast
Source: EMBO J. 2024 Sep 10;43(20):9. doi: 10.1038/s44318-024-00224-z (PMC11480445; doi:10.1038/s44318-024-00224-z)

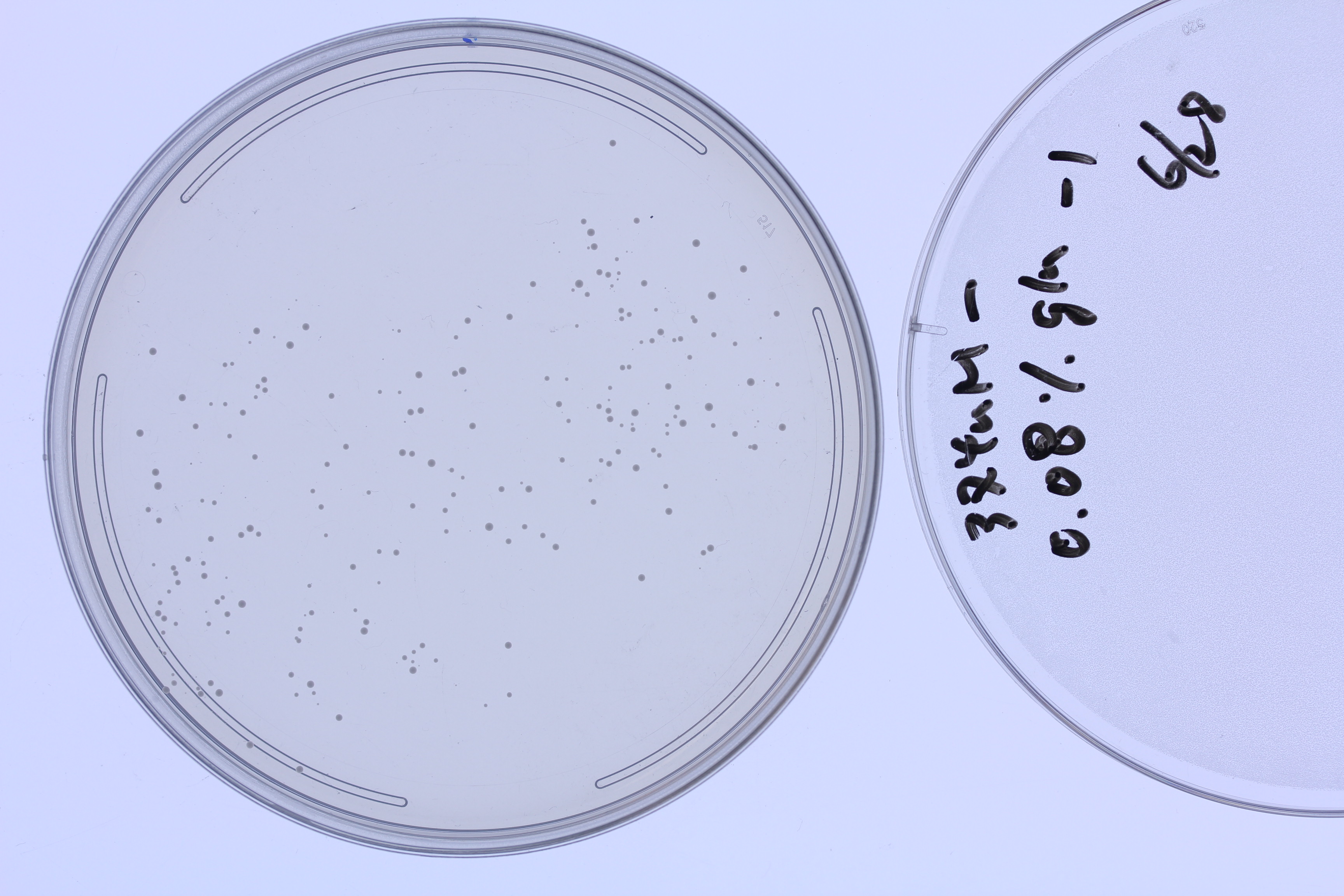

Supplement: Supplementary file 8 — Source data Fig. 1 [file 44318_2024_224_MOESM8_ESM.zip › EMBOJ-2024-117143-T-R_SourceData_Figure 1/ImageData/1F/374mMNH4Cl_0.08%glu_14day.tif]

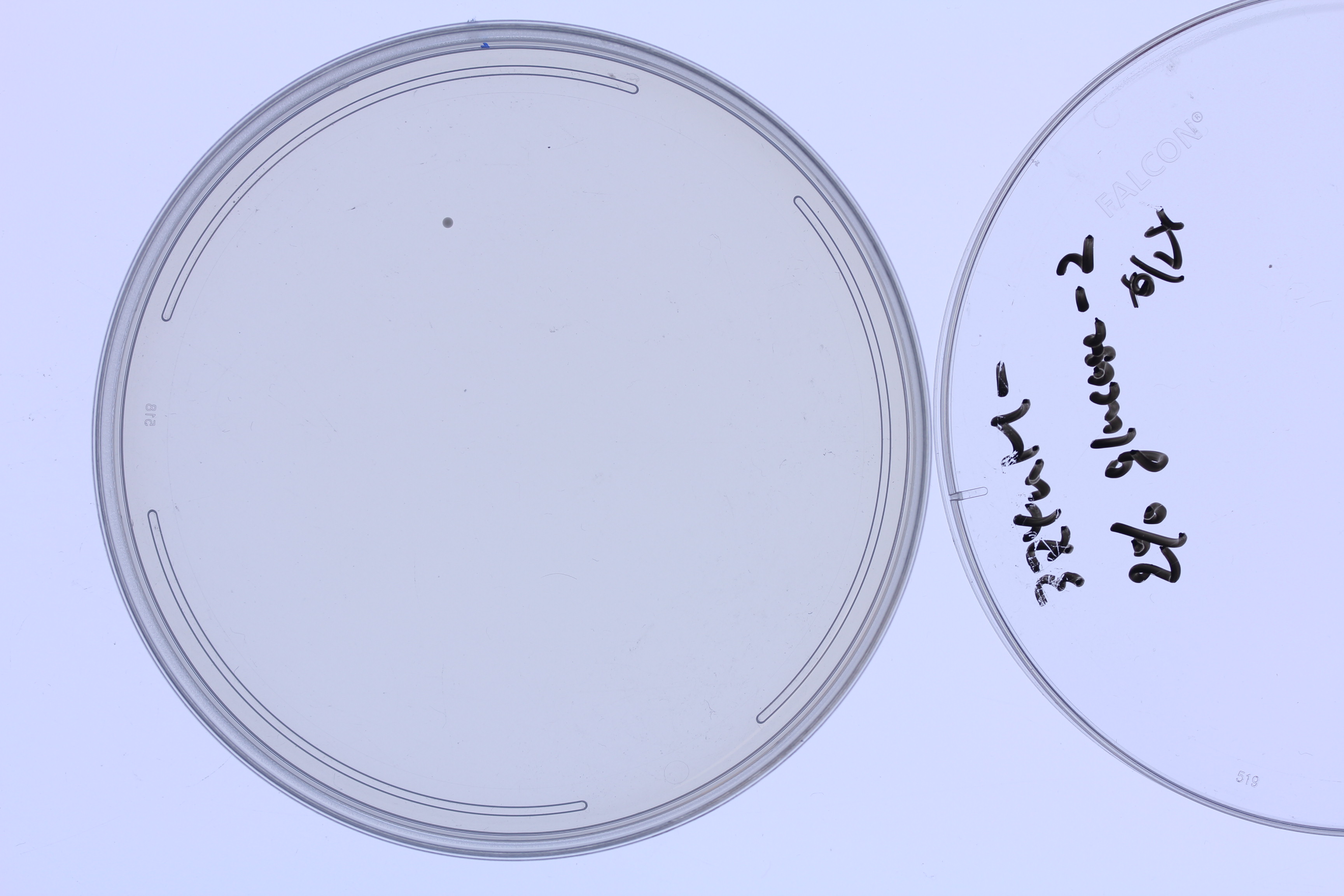

Supplement: Supplementary file 8 — Source data Fig. 1 [file 44318_2024_224_MOESM8_ESM.zip › EMBOJ-2024-117143-T-R_SourceData_Figure 1/ImageData/1F/374mMNH4Cl_2%glu_14day.tif]

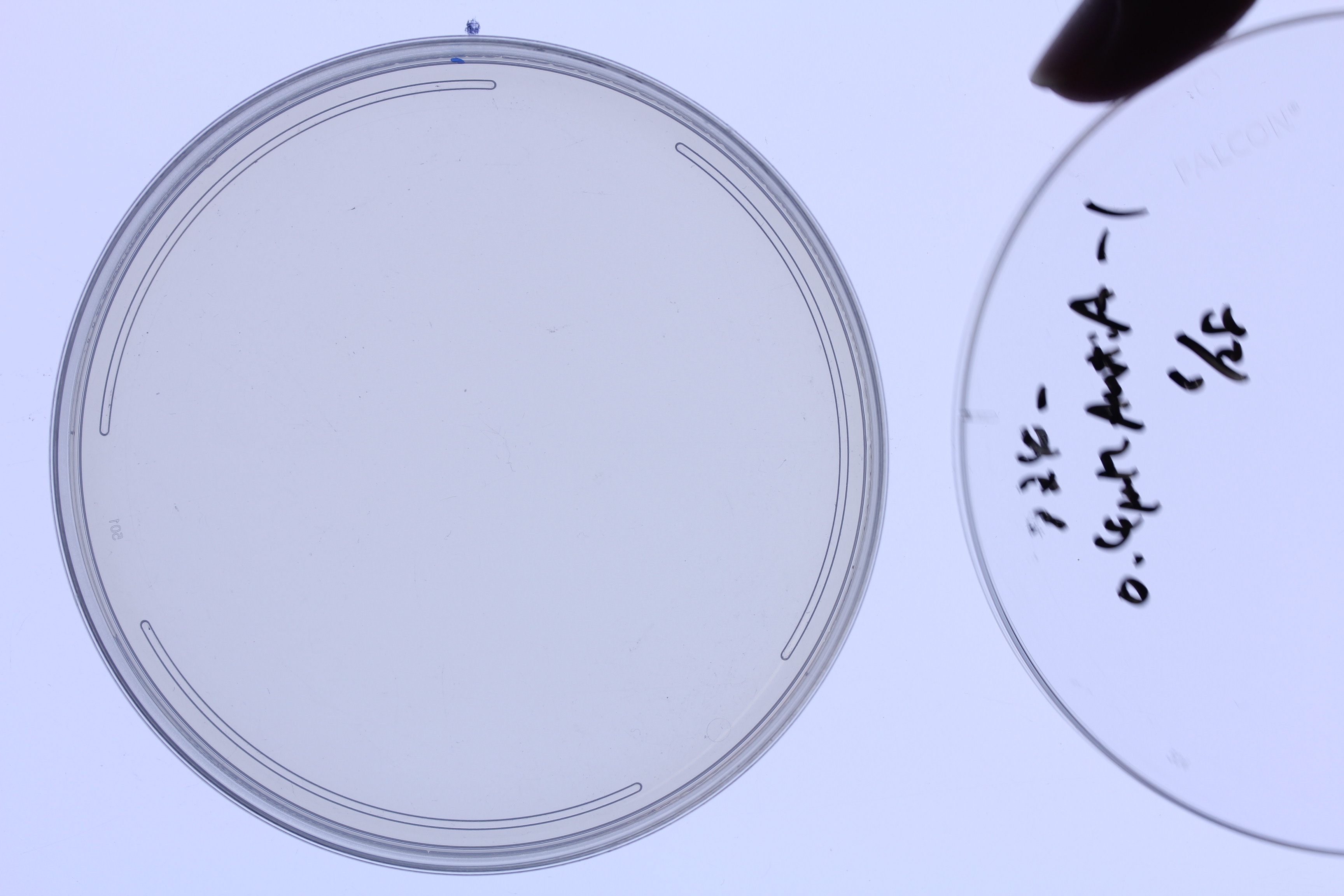

Supplement: Supplementary file 8 — Source data Fig. 1 [file 44318_2024_224_MOESM8_ESM.zip › EMBOJ-2024-117143-T-R_SourceData_Figure 1/ImageData/1E/374mMNH4Cl_0.4uMantiA_14day.tif]

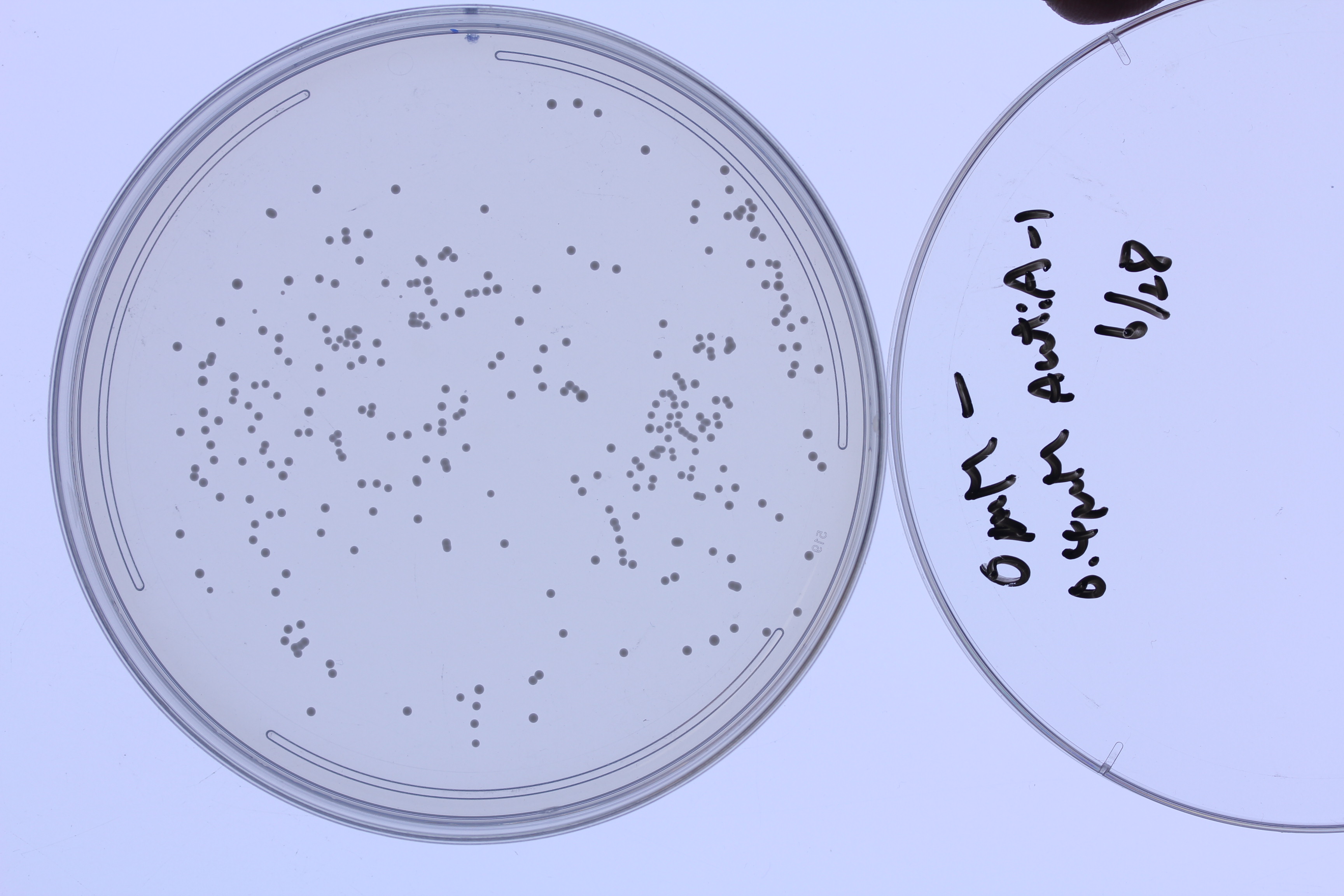

Supplement: Supplementary file 8 — Source data Fig. 1 [file 44318_2024_224_MOESM8_ESM.zip › EMBOJ-2024-117143-T-R_SourceData_Figure 1/ImageData/1E/0mMNH4Cl_0.4uMantiA_5day.tif]

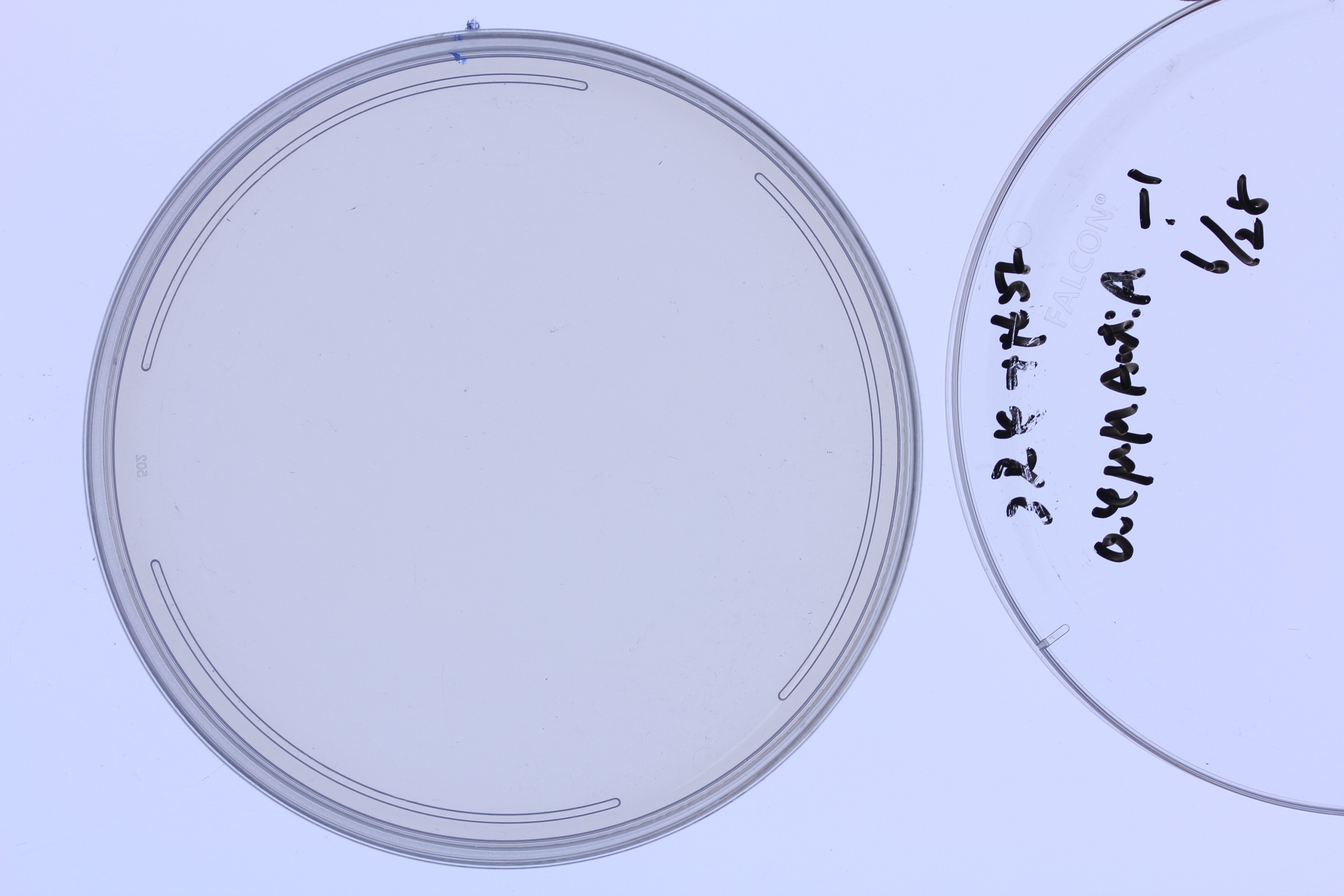

Supplement: Supplementary file 8 — Source data Fig. 1 [file 44318_2024_224_MOESM8_ESM.zip › EMBOJ-2024-117143-T-R_SourceData_Figure 1/ImageData/1E/374mMNH4Cl_0.4uMantiA_NSF_14day.tif]

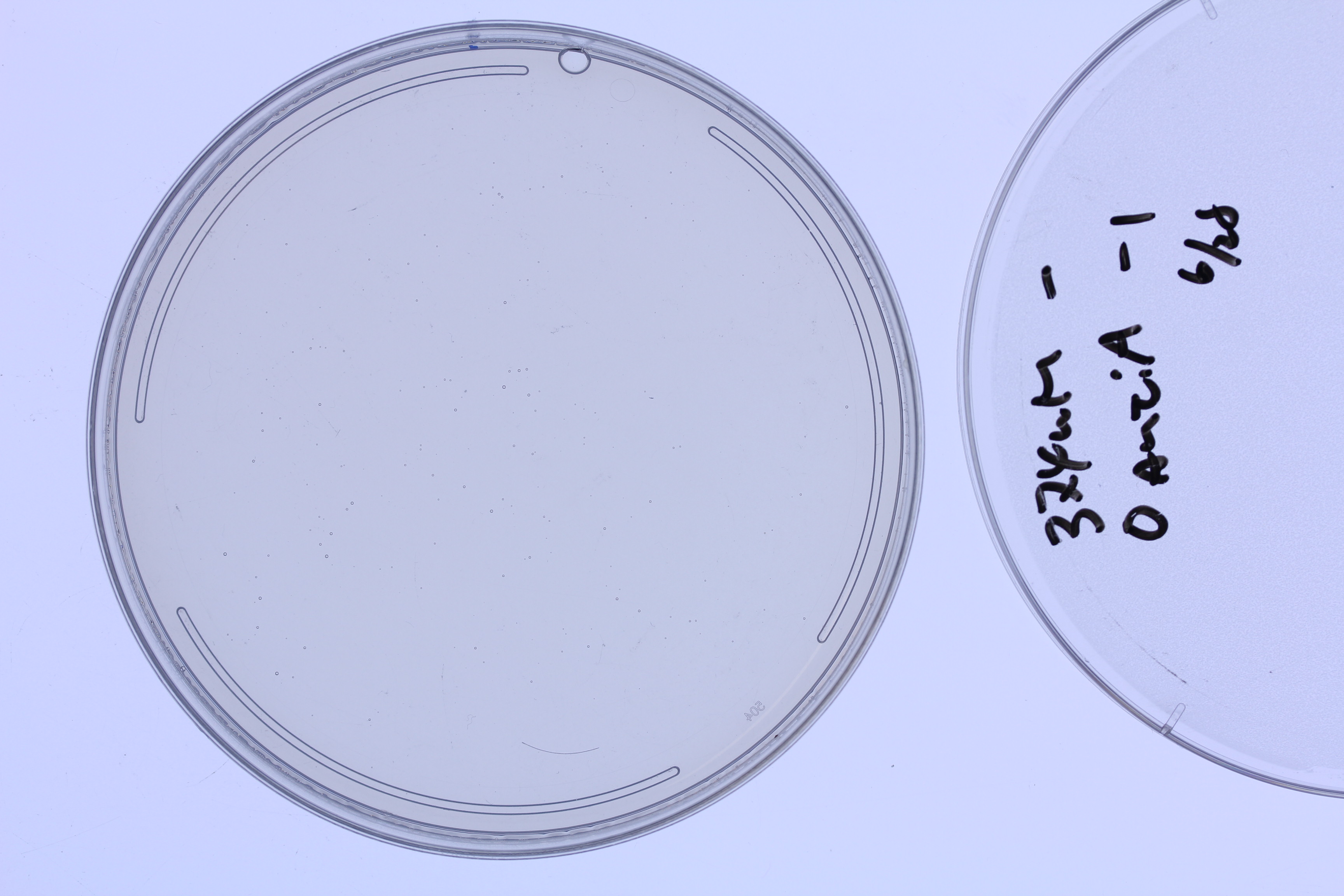

Supplement: Supplementary file 8 — Source data Fig. 1 [file 44318_2024_224_MOESM8_ESM.zip › EMBOJ-2024-117143-T-R_SourceData_Figure 1/ImageData/1E/374mMNH4Cl_0uMantiA_5day.tif]

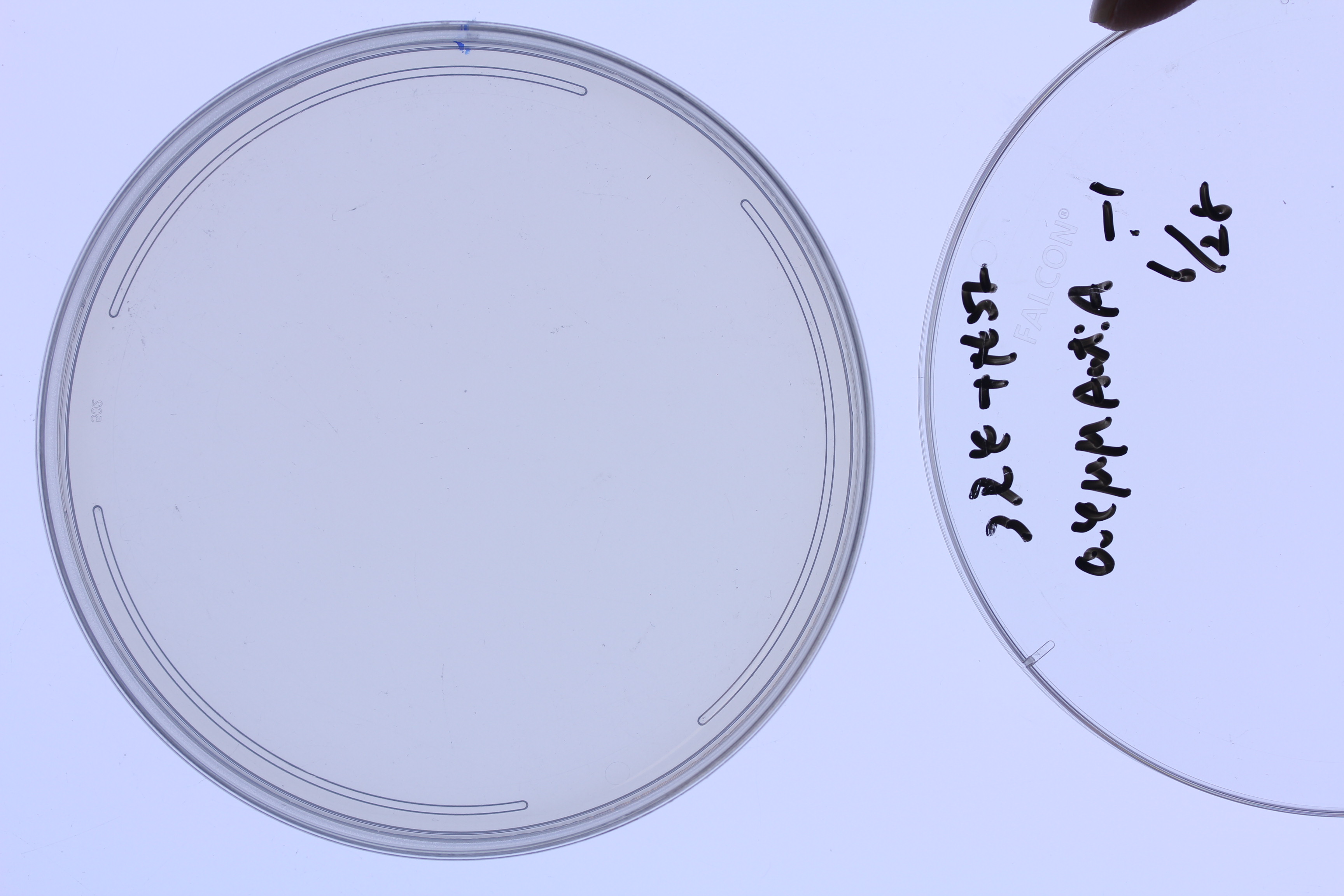

Supplement: Supplementary file 8 — Source data Fig. 1 [file 44318_2024_224_MOESM8_ESM.zip › EMBOJ-2024-117143-T-R_SourceData_Figure 1/ImageData/1E/374mMNH4Cl_0.4uMantiA_NSF_5day.tif]

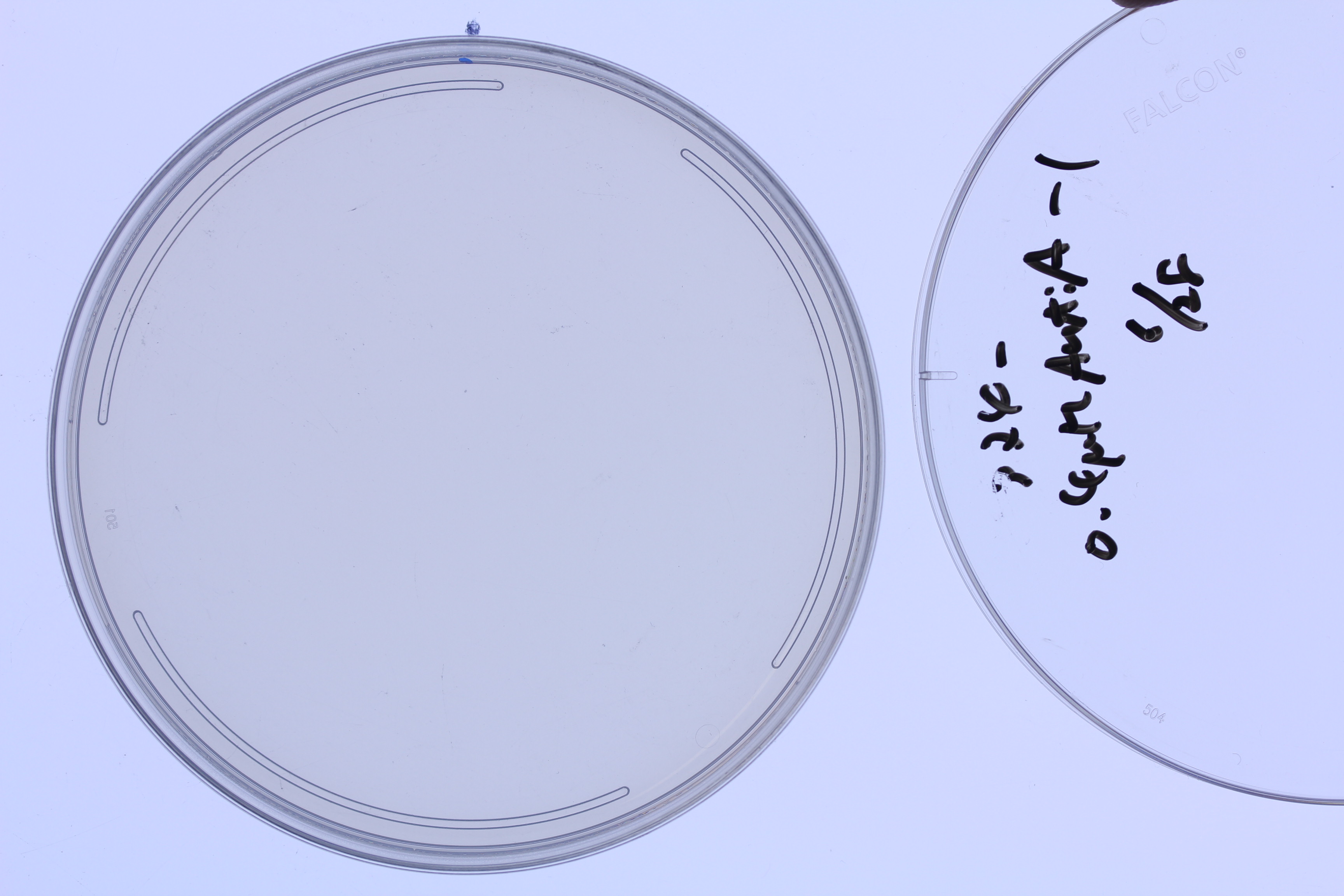

Supplement: Supplementary file 8 — Source data Fig. 1 [file 44318_2024_224_MOESM8_ESM.zip › EMBOJ-2024-117143-T-R_SourceData_Figure 1/ImageData/1E/374mMNH4Cl_0.4uMantiA_5day.tif]

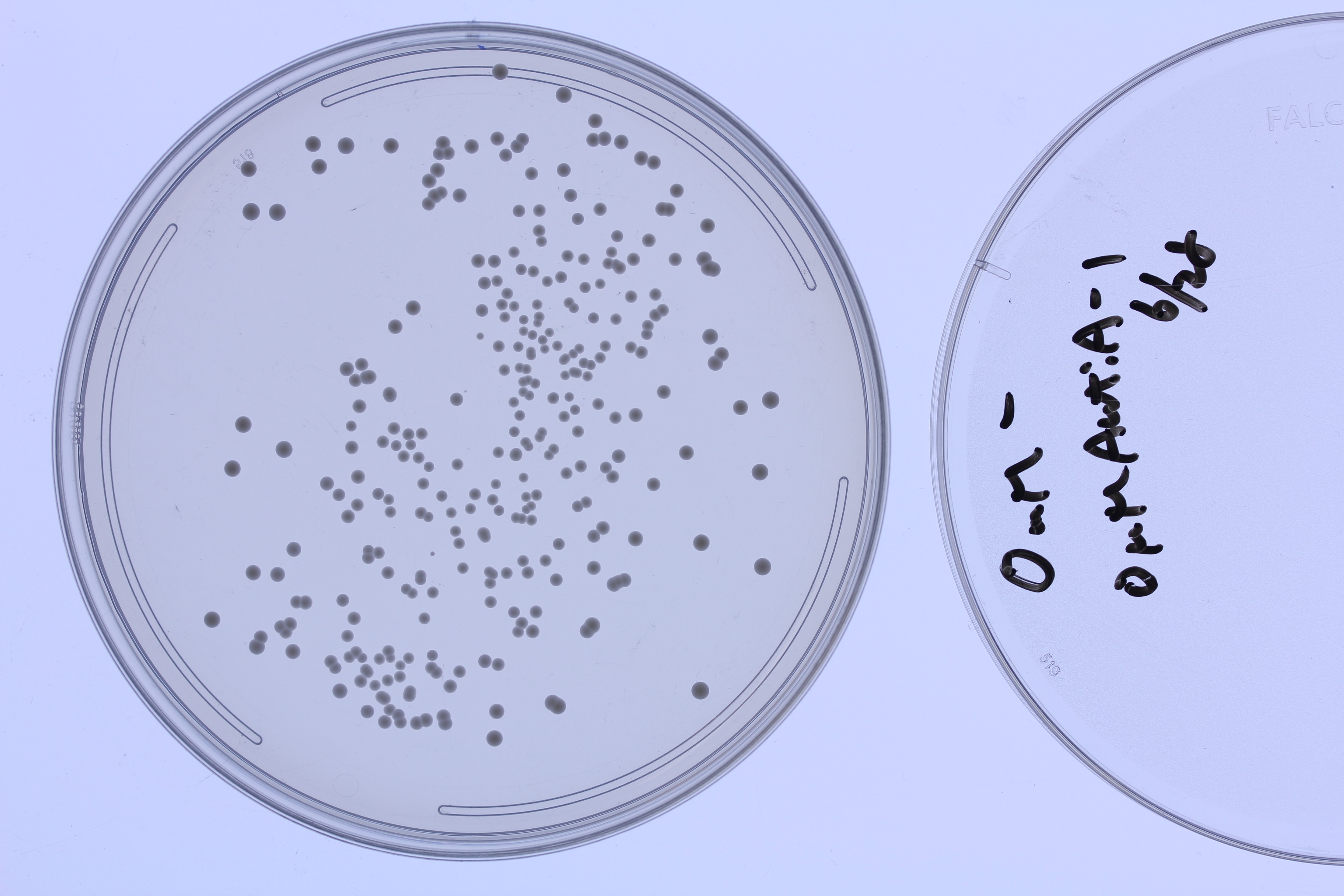

Supplement: Supplementary file 8 — Source data Fig. 1 [file 44318_2024_224_MOESM8_ESM.zip › EMBOJ-2024-117143-T-R_SourceData_Figure 1/ImageData/1E/0mMNH4Cl_0uMantiA_5day.tif]

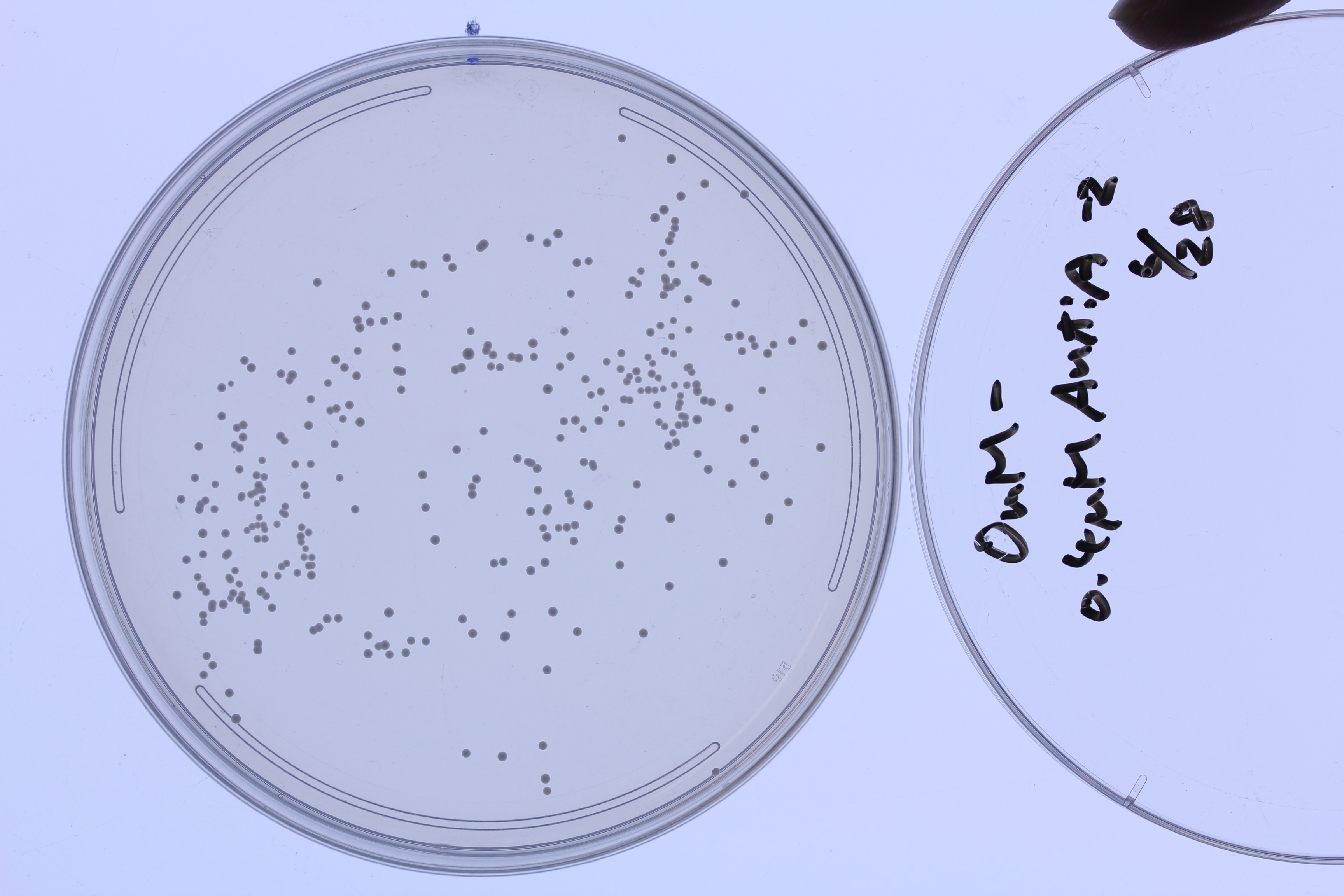

Supplement: Supplementary file 8 — Source data Fig. 1 [file 44318_2024_224_MOESM8_ESM.zip › EMBOJ-2024-117143-T-R_SourceData_Figure 1/ImageData/1E/0mMNH4Cl_0.4uMantiA_NSF_5day.tif]

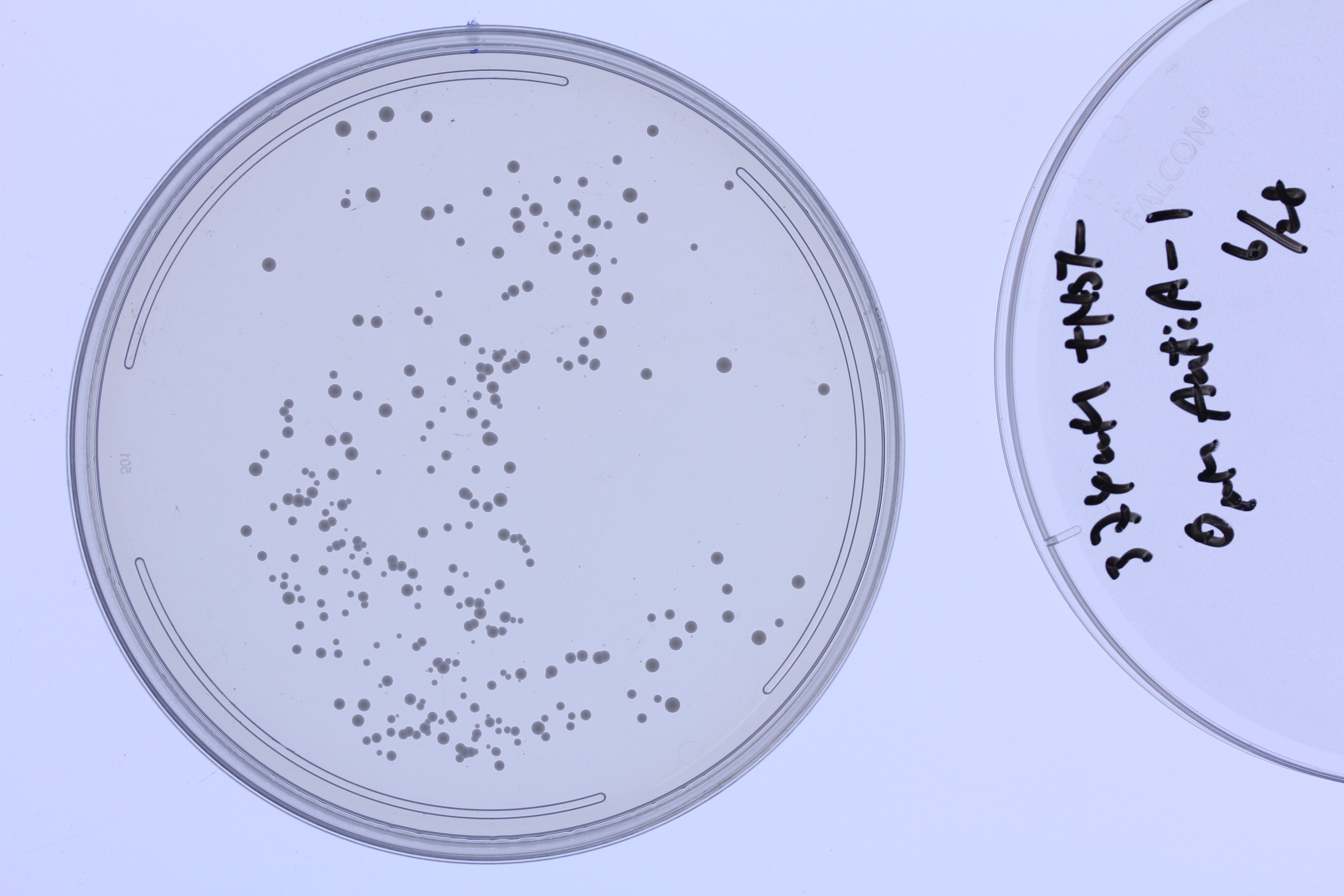

Supplement: Supplementary file 8 — Source data Fig. 1 [file 44318_2024_224_MOESM8_ESM.zip › EMBOJ-2024-117143-T-R_SourceData_Figure 1/ImageData/1E/374mMNH4Cl_0uMantiA_NSF_5day.tif]

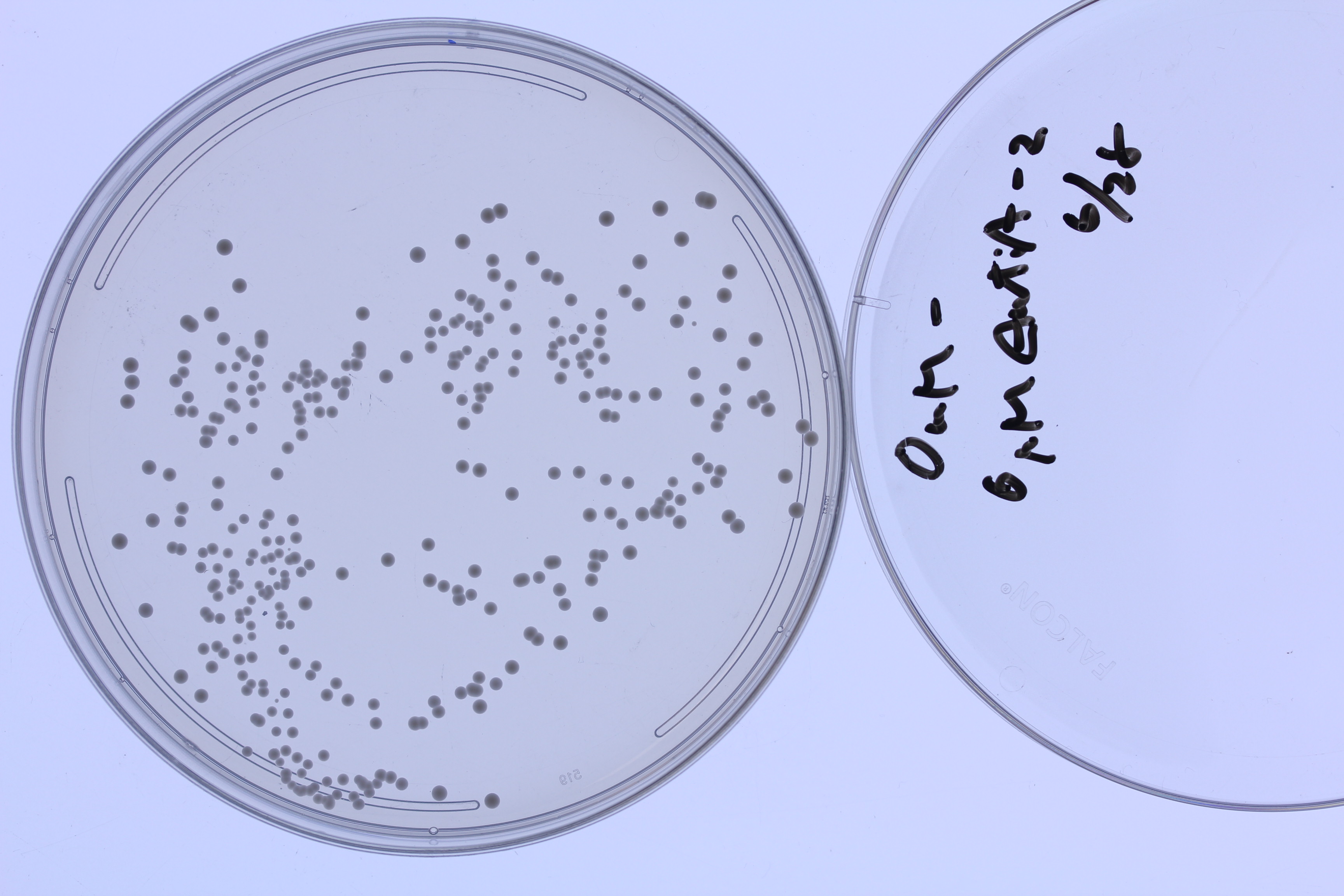

Supplement: Supplementary file 8 — Source data Fig. 1 [file 44318_2024_224_MOESM8_ESM.zip › EMBOJ-2024-117143-T-R_SourceData_Figure 1/ImageData/1E/0mMNH4Cl_0uMantiA_NSF_5day.tif]

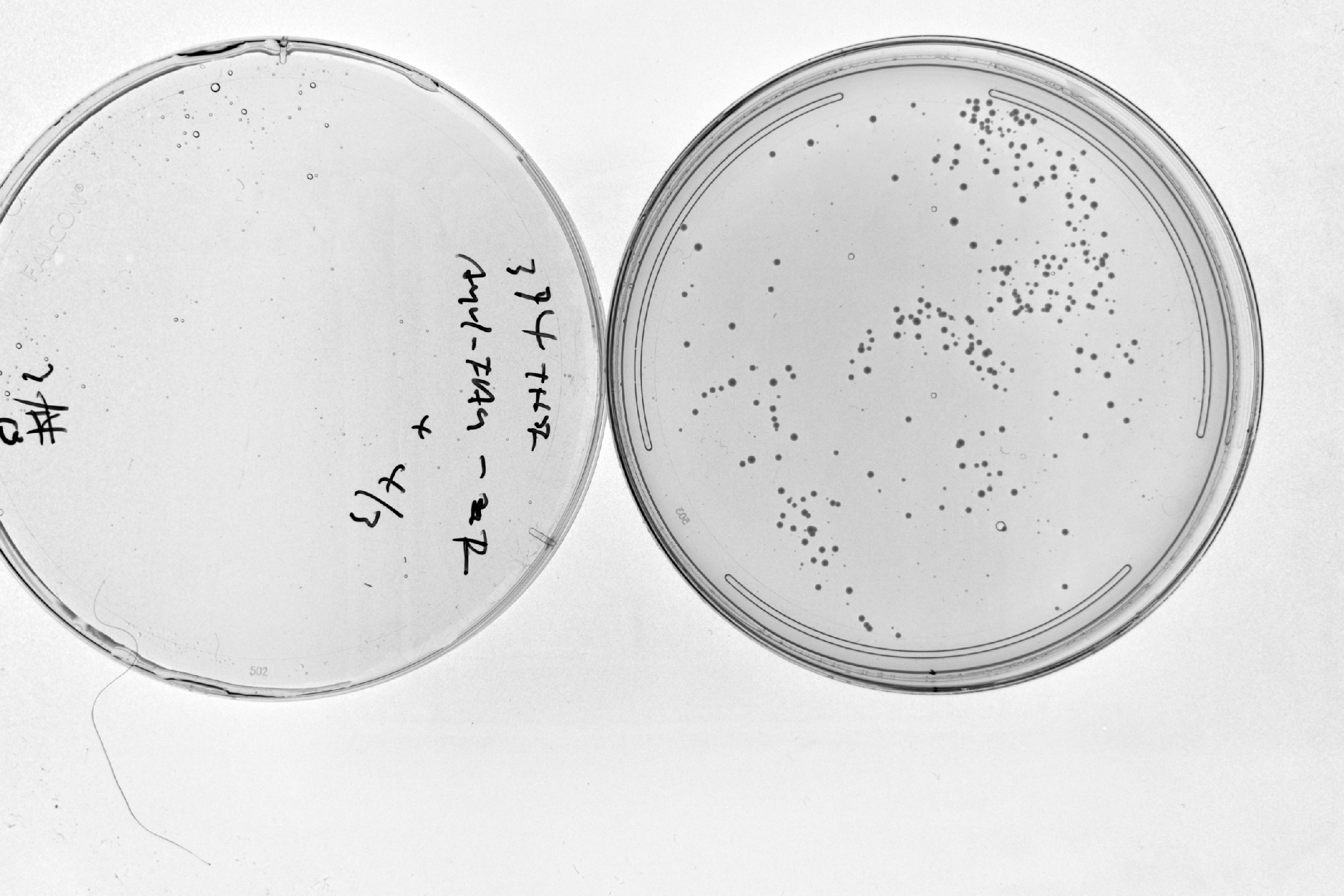

Supplement: Supplementary file 11 — Source data Fig. 5 [file 44318_2024_224_MOESM11_ESM.zip › EMBOJ-2024-117143-T-R_SourceData_Figure 5/ImageData/5F/374mMNH4Cl_Ayr1-FLAG_NSF_5day.tif]

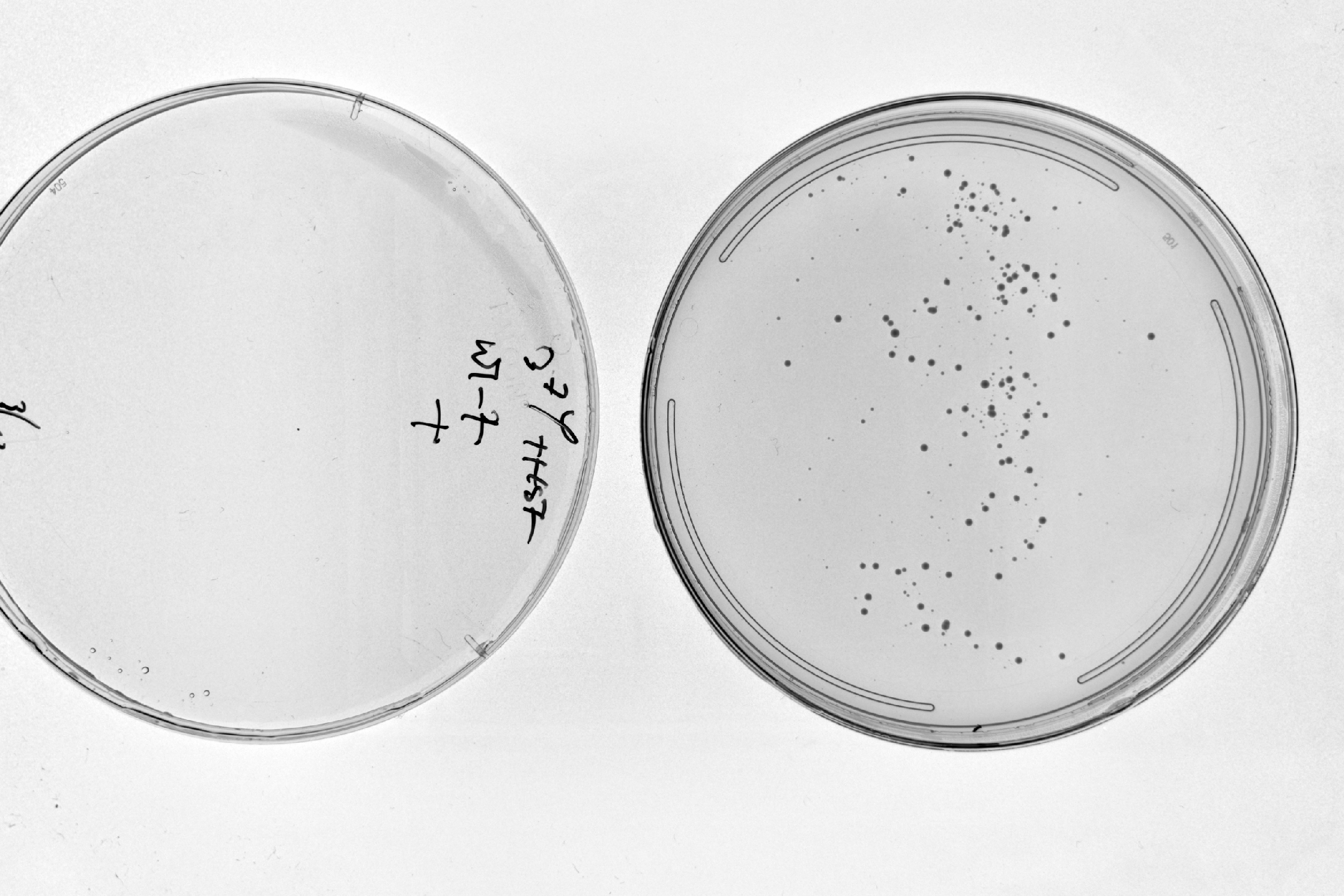

Supplement: Supplementary file 11 — Source data Fig. 5 [file 44318_2024_224_MOESM11_ESM.zip › EMBOJ-2024-117143-T-R_SourceData_Figure 5/ImageData/5F/374mMNH4Cl_WT_NSF_5day.tif.tif]

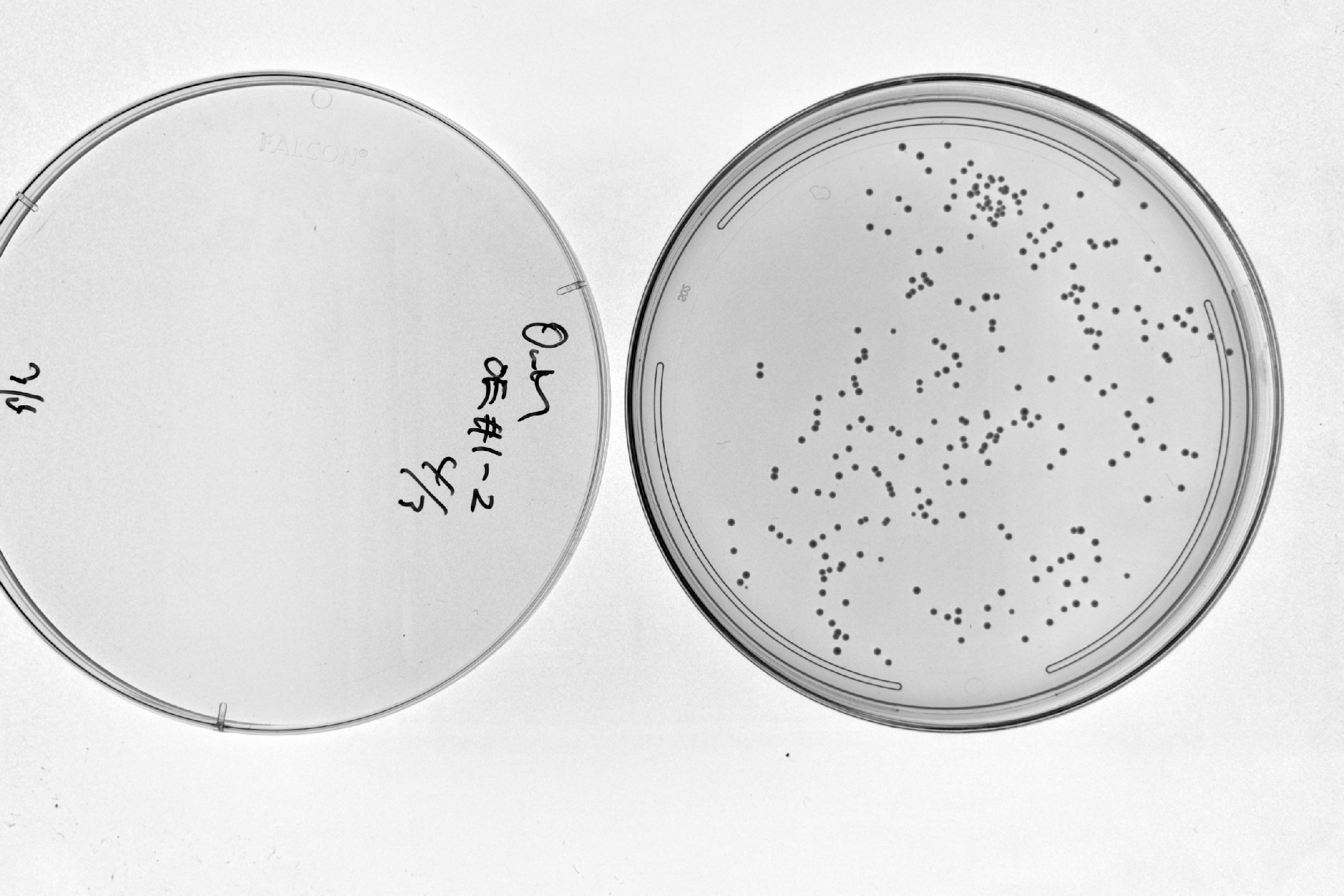

Supplement: Supplementary file 11 — Source data Fig. 5 [file 44318_2024_224_MOESM11_ESM.zip › EMBOJ-2024-117143-T-R_SourceData_Figure 5/ImageData/5F/0mMNH4Cl_Ayr1OE#2_5day.tif]

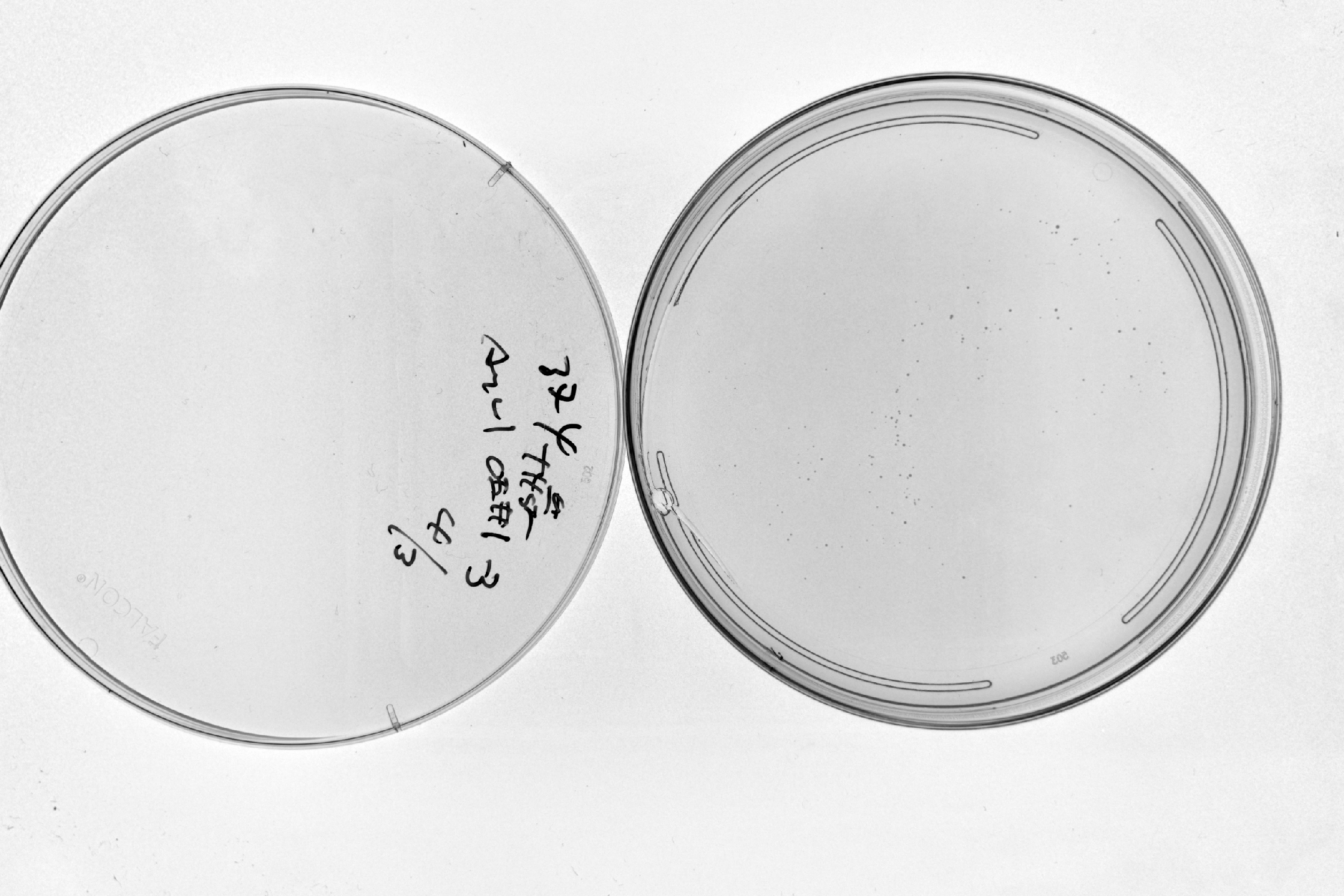

Supplement: Supplementary file 11 — Source data Fig. 5 [file 44318_2024_224_MOESM11_ESM.zip › EMBOJ-2024-117143-T-R_SourceData_Figure 5/ImageData/5F/374mMNH4Cl_Ayr1OE#2_NSF_5day.tif]

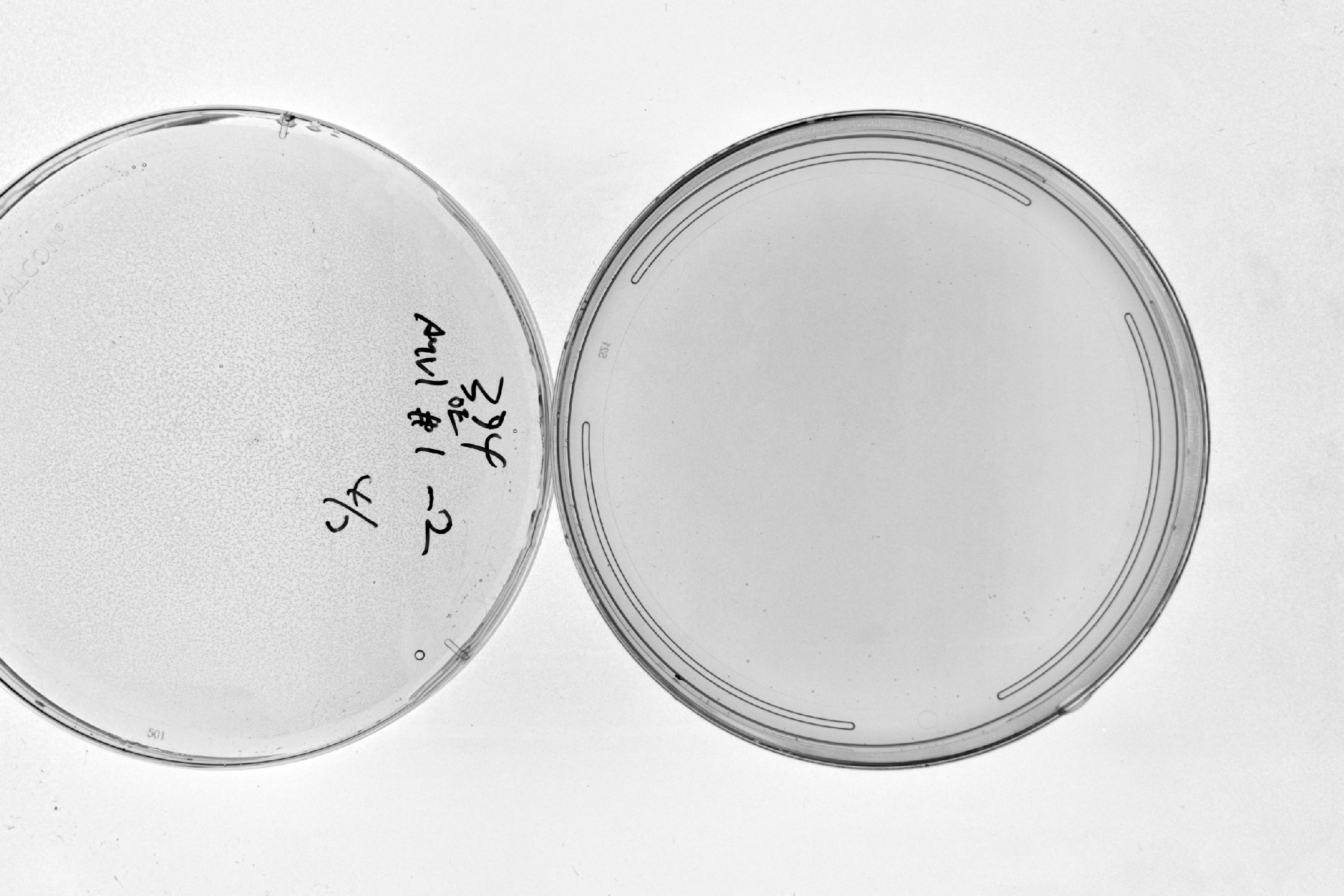

Supplement: Supplementary file 11 — Source data Fig. 5 [file 44318_2024_224_MOESM11_ESM.zip › EMBOJ-2024-117143-T-R_SourceData_Figure 5/ImageData/5F/374mMNH4Cl_Ayr1OE#1_5day.tif]

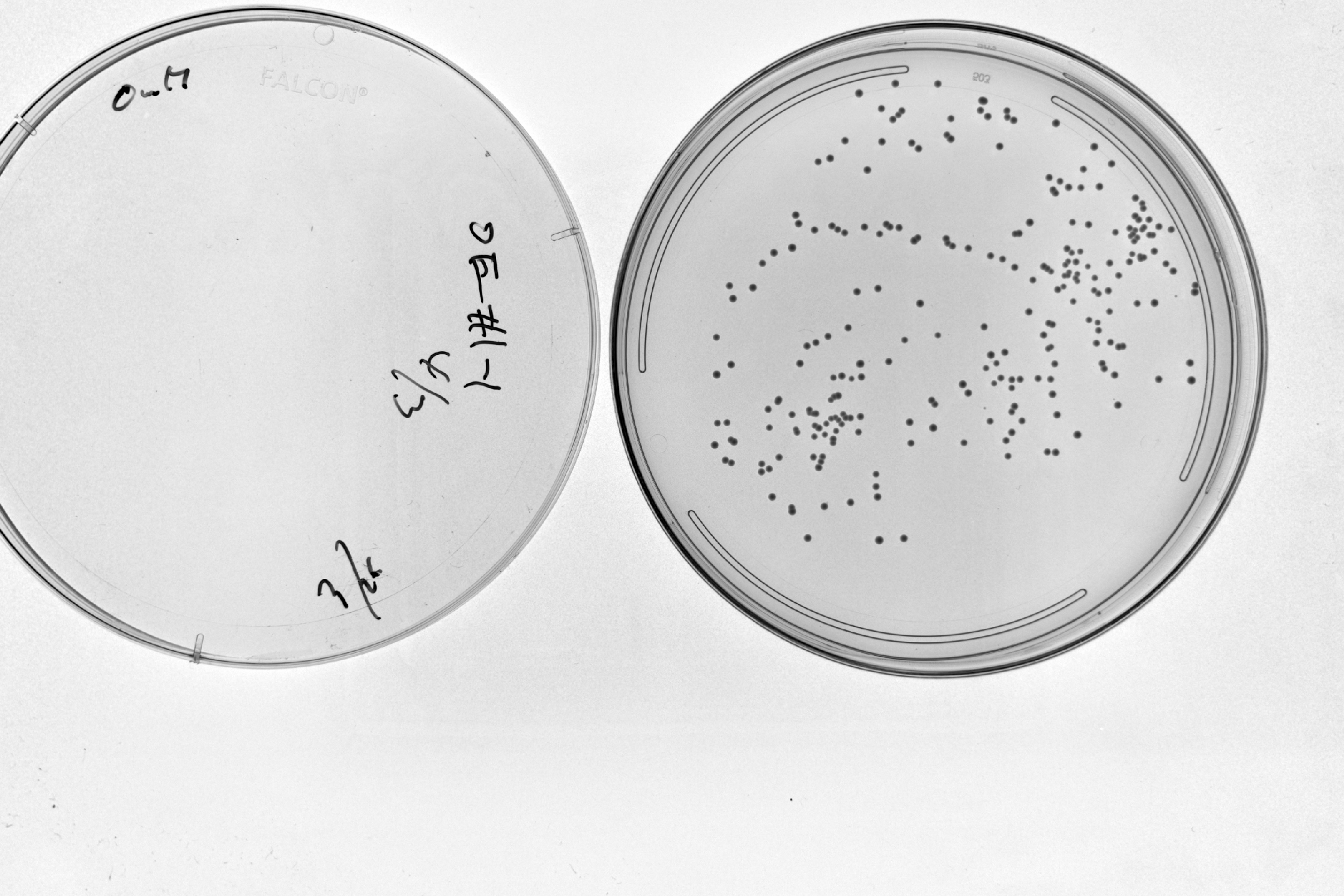

Supplement: Supplementary file 11 — Source data Fig. 5 [file 44318_2024_224_MOESM11_ESM.zip › EMBOJ-2024-117143-T-R_SourceData_Figure 5/ImageData/5F/0mMNH4Cl_Ayr1OE#1_5day.tif]

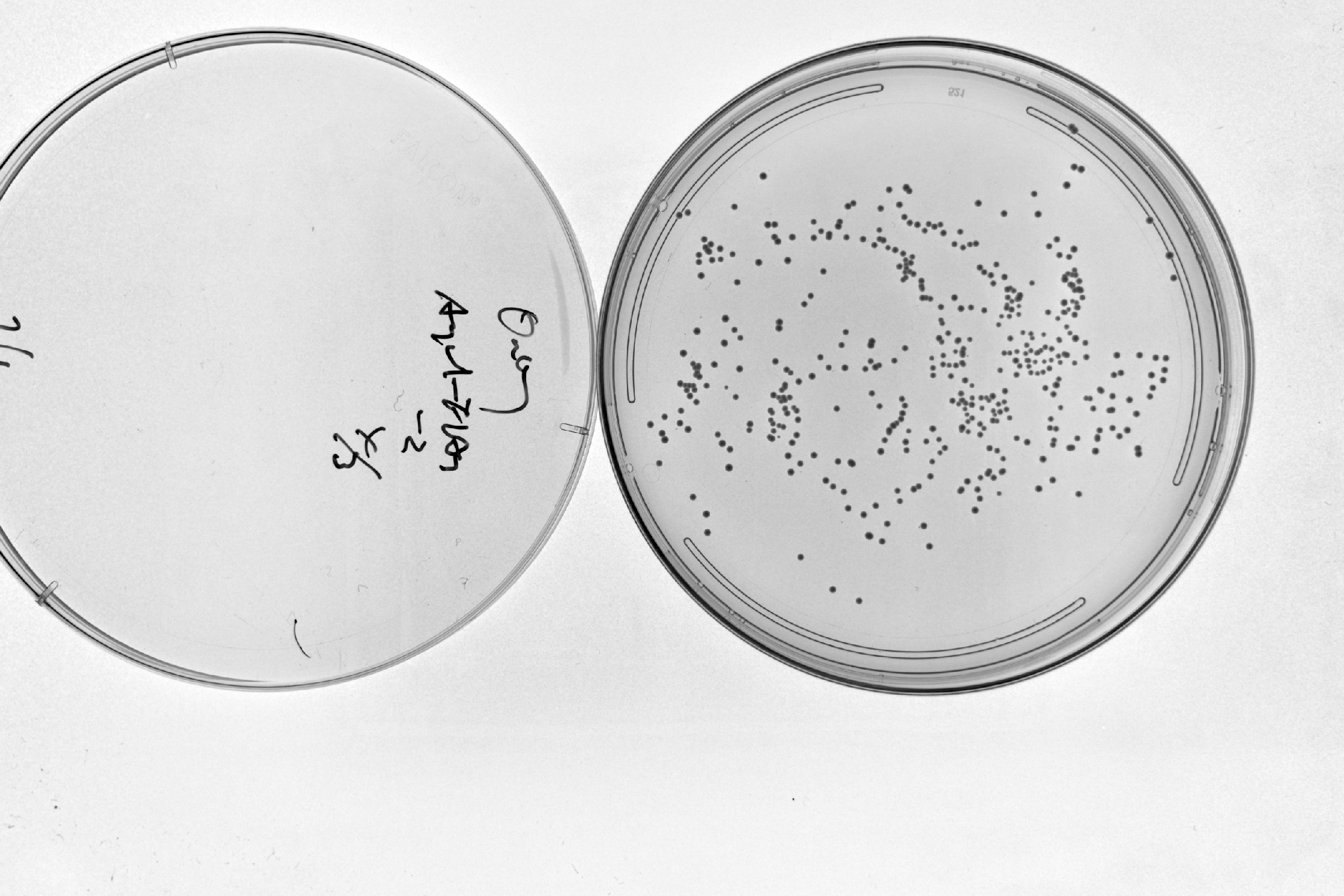

Supplement: Supplementary file 11 — Source data Fig. 5 [file 44318_2024_224_MOESM11_ESM.zip › EMBOJ-2024-117143-T-R_SourceData_Figure 5/ImageData/5F/0mMNH4Cl_Ayr1-FLAG_5day.tif]

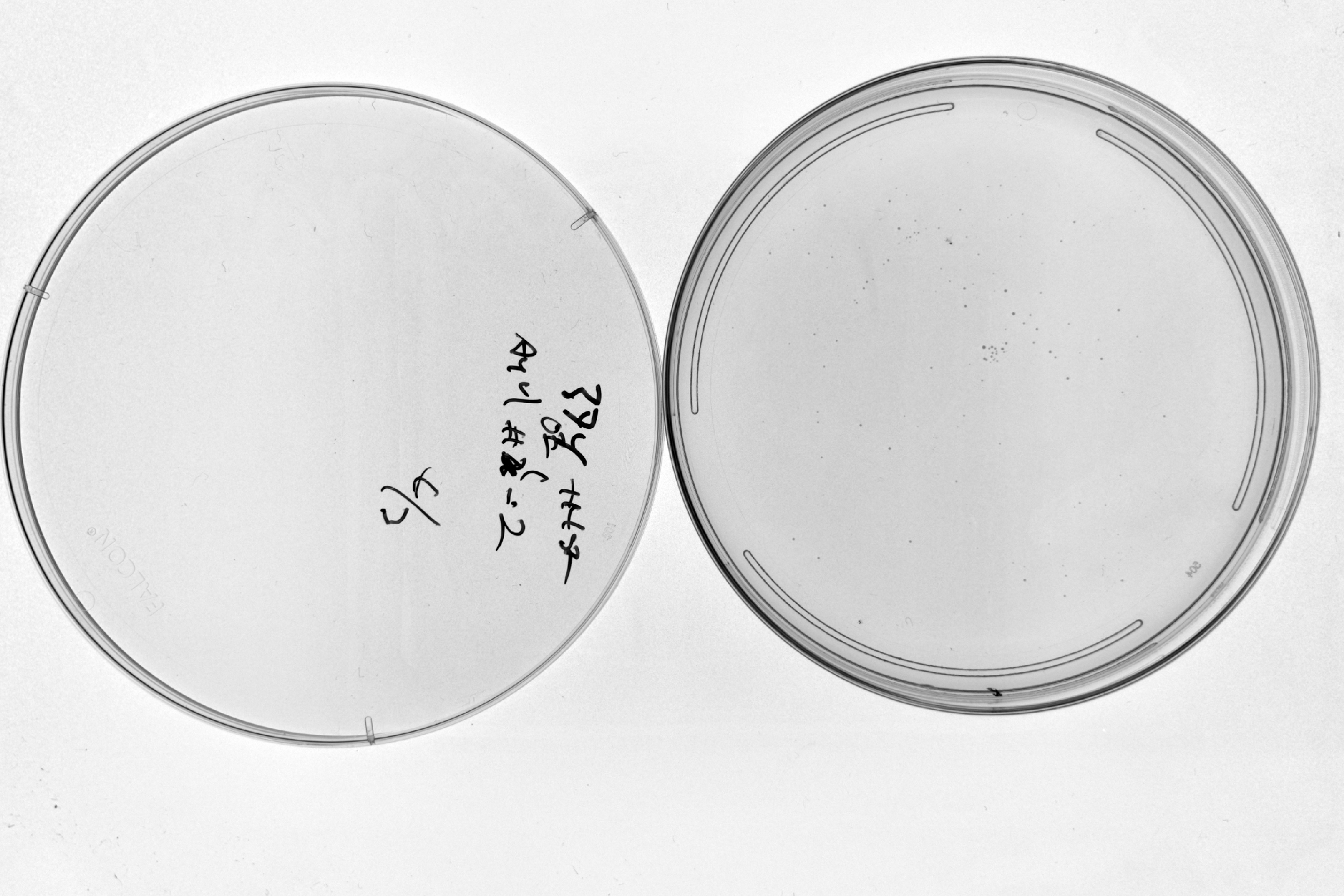

Supplement: Supplementary file 11 — Source data Fig. 5 [file 44318_2024_224_MOESM11_ESM.zip › EMBOJ-2024-117143-T-R_SourceData_Figure 5/ImageData/5F/374mMNH4Cl_Ayr1OE#1_NSF_5day.tif]

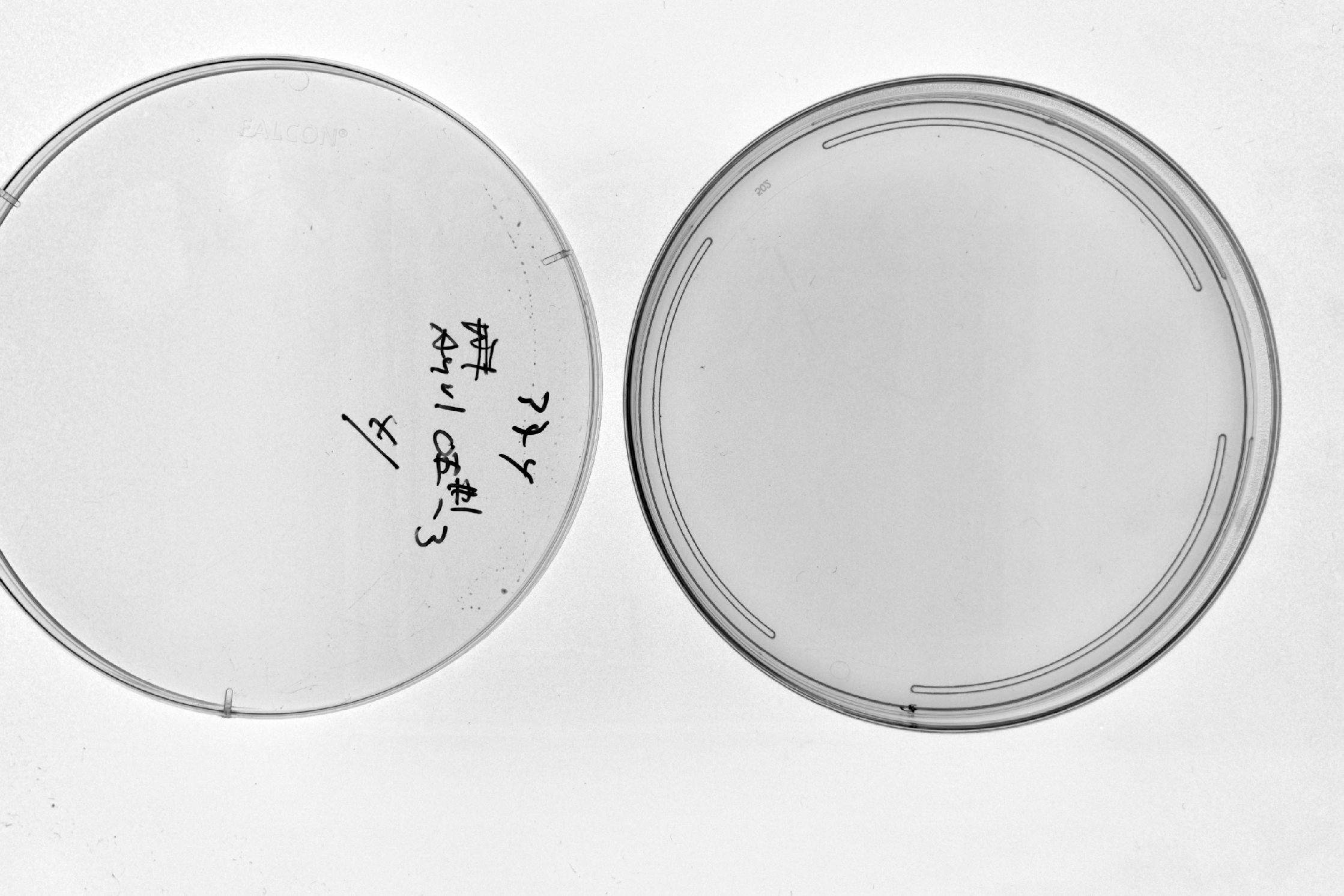

Supplement: Supplementary file 11 — Source data Fig. 5 [file 44318_2024_224_MOESM11_ESM.zip › EMBOJ-2024-117143-T-R_SourceData_Figure 5/ImageData/5F/374mMNH4Cl_Ayr1OE#2_5day.tif]

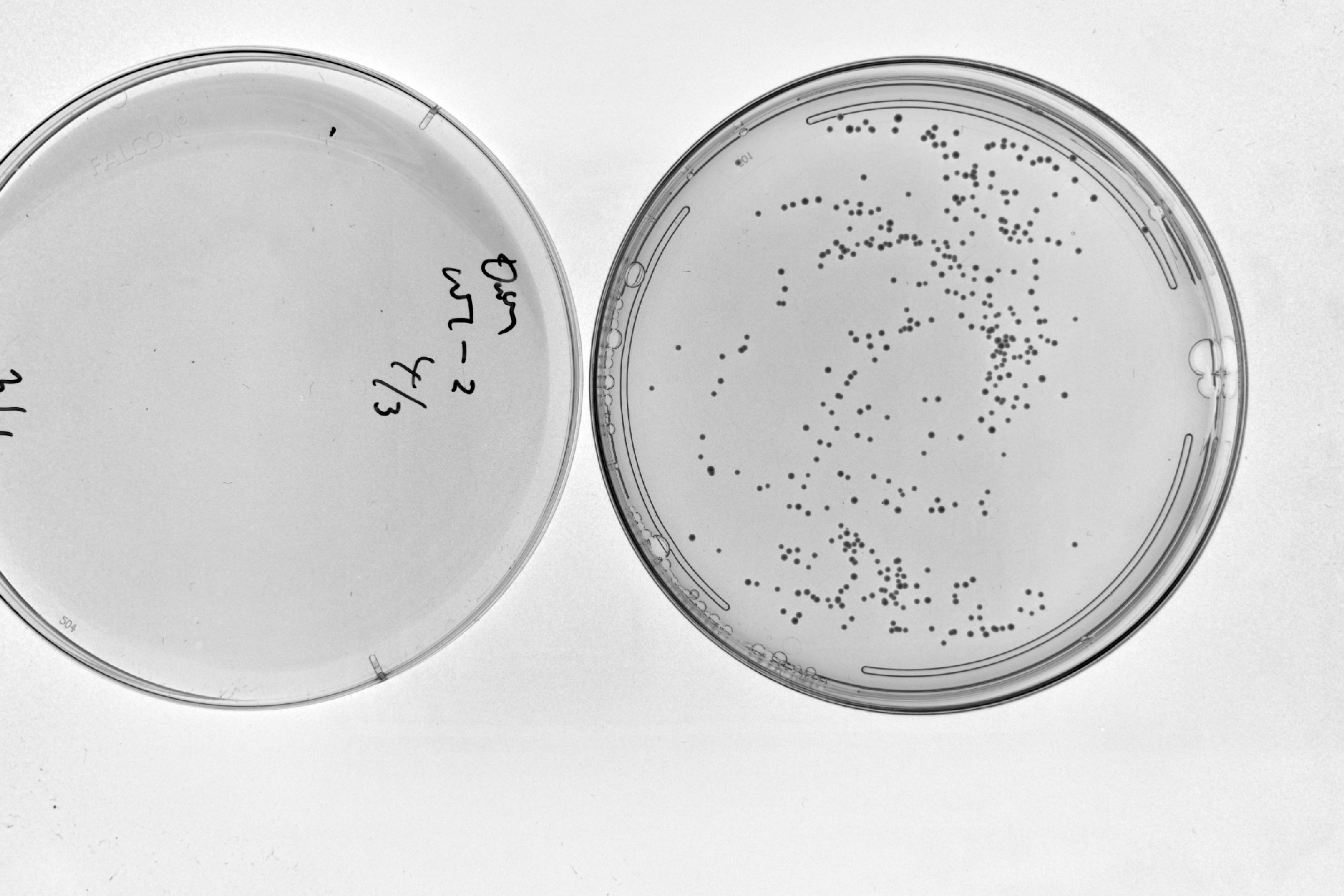

Supplement: Supplementary file 11 — Source data Fig. 5 [file 44318_2024_224_MOESM11_ESM.zip › EMBOJ-2024-117143-T-R_SourceData_Figure 5/ImageData/5F/0mMNH4Cl_WT_5day.tif]

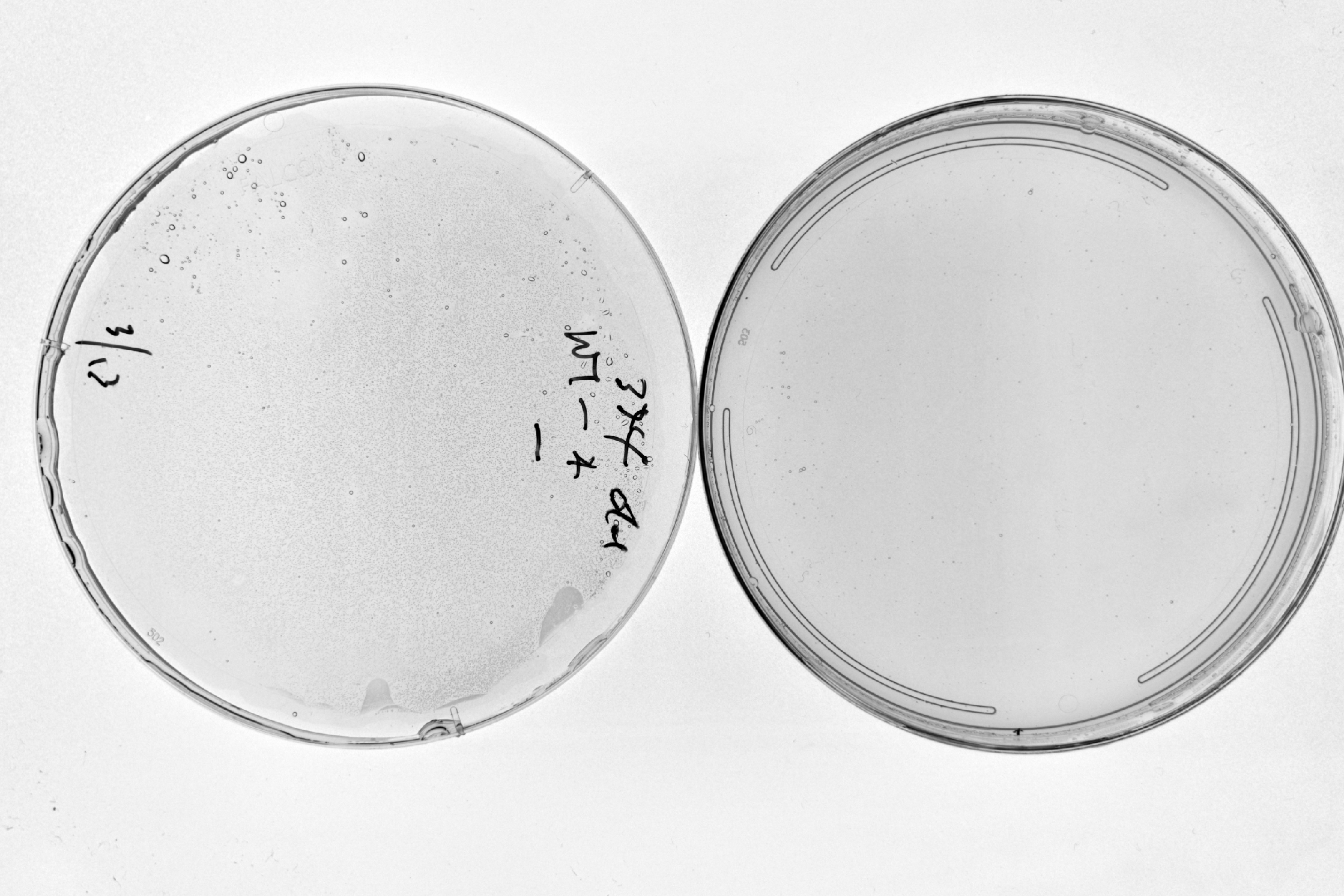

Supplement: Supplementary file 11 — Source data Fig. 5 [file 44318_2024_224_MOESM11_ESM.zip › EMBOJ-2024-117143-T-R_SourceData_Figure 5/ImageData/5F/374mMNH4Cl_WT_5day.tif]

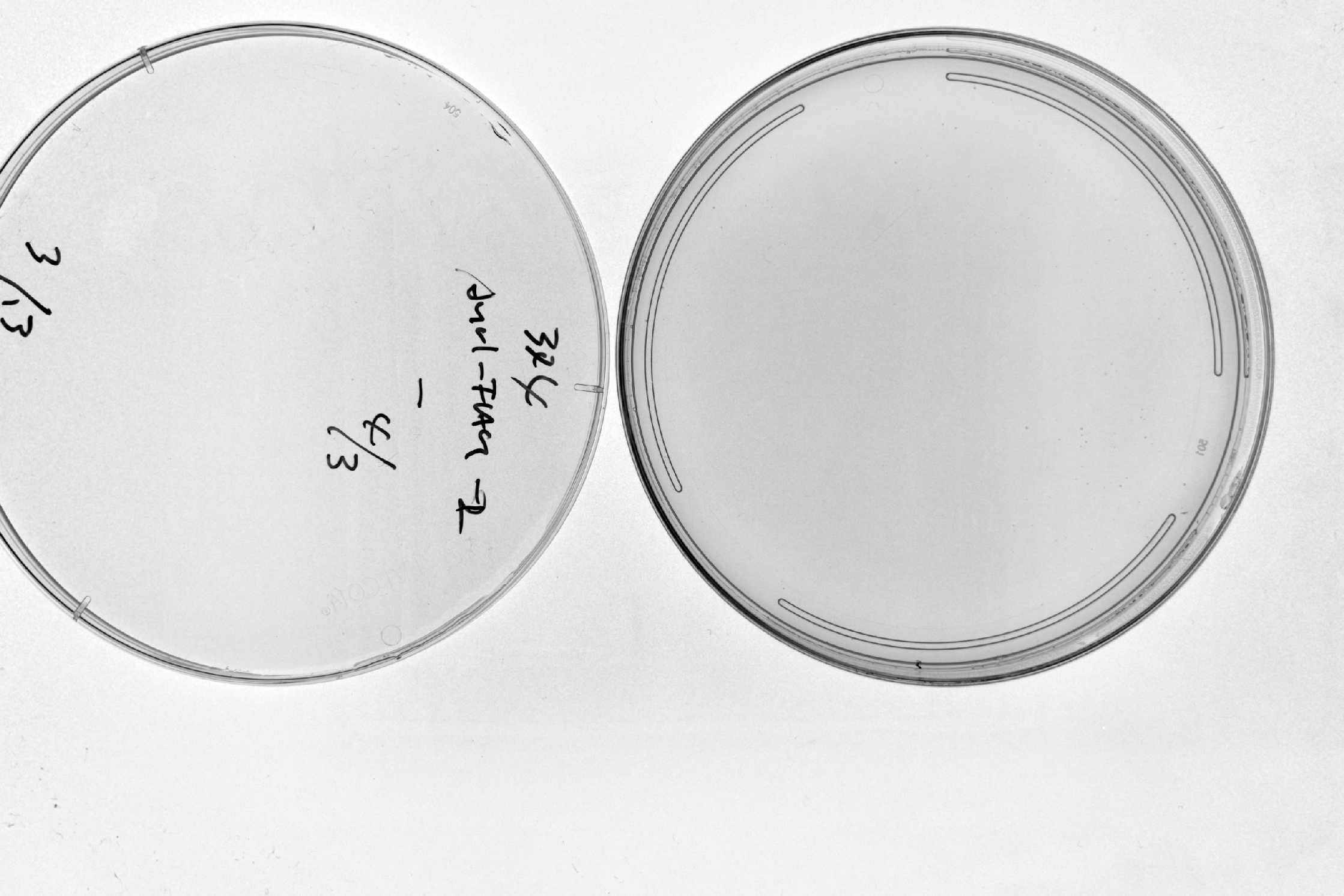

Supplement: Supplementary file 11 — Source data Fig. 5 [file 44318_2024_224_MOESM11_ESM.zip › EMBOJ-2024-117143-T-R_SourceData_Figure 5/ImageData/5F/374mMNH4Cl_Ayr1-FLAG_5day.tif.tif]

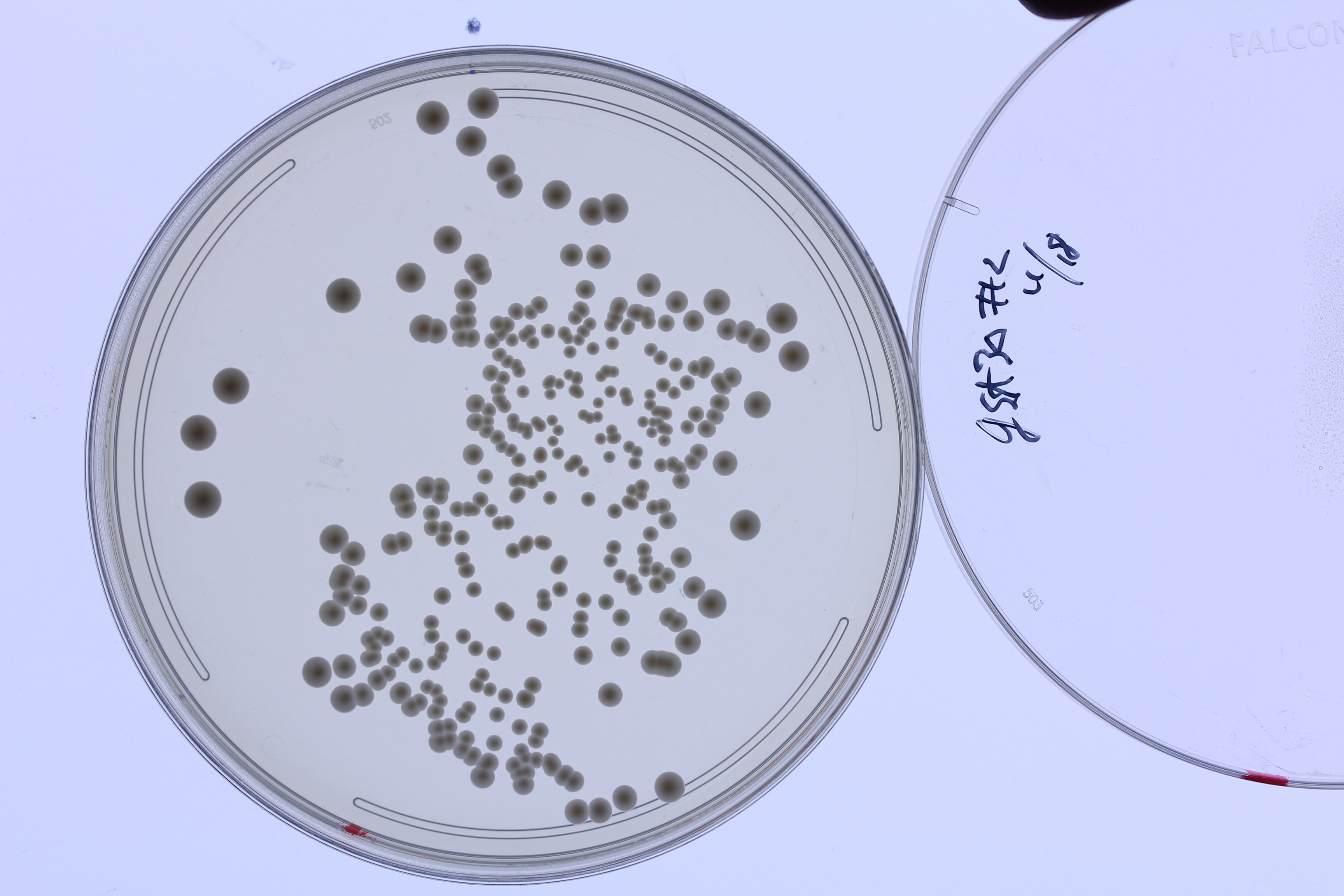

Supplement: Supplementary file 11 — Source data Fig. 5 [file 44318_2024_224_MOESM11_ESM.zip › EMBOJ-2024-117143-T-R_SourceData_Figure 5/ImageData/5J/YES_gst3d_5day.tif]

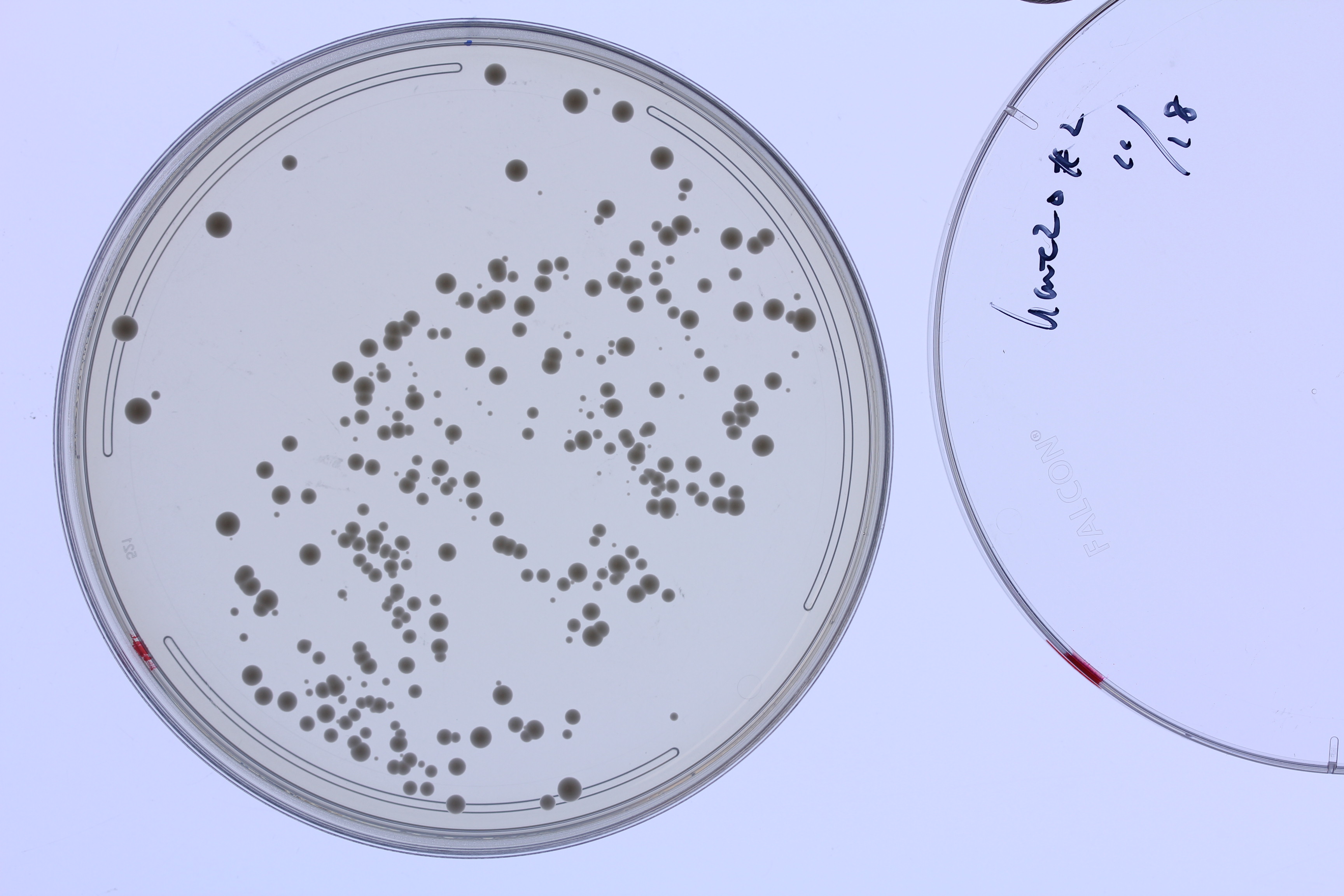

Supplement: Supplementary file 11 — Source data Fig. 5 [file 44318_2024_224_MOESM11_ESM.zip › EMBOJ-2024-117143-T-R_SourceData_Figure 5/ImageData/5J/YES_hmt2d_5day.tif]

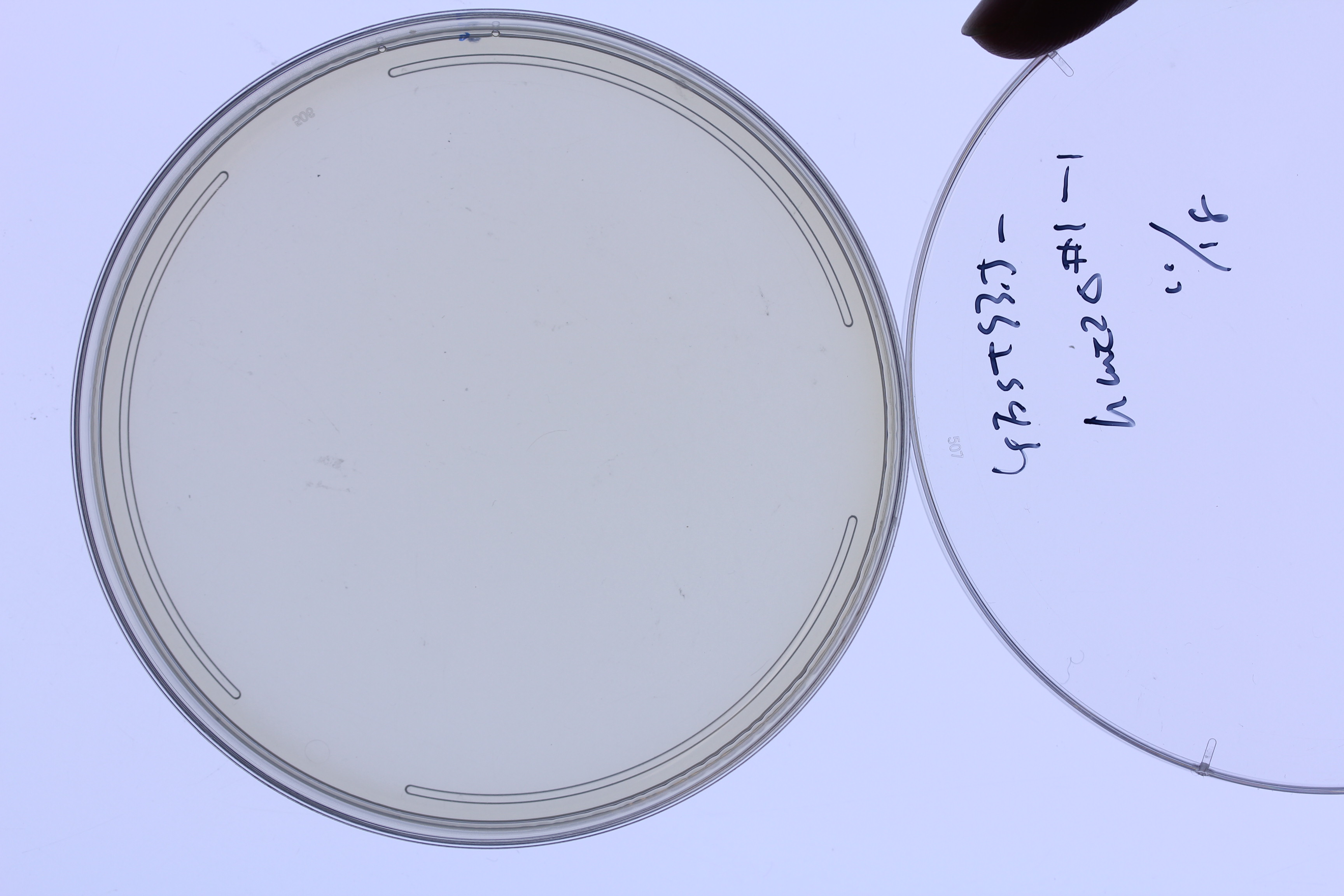

Supplement: Supplementary file 11 — Source data Fig. 5 [file 44318_2024_224_MOESM11_ESM.zip › EMBOJ-2024-117143-T-R_SourceData_Figure 5/ImageData/5J/YES93NH4Cl_hmt2d_10day.tif]

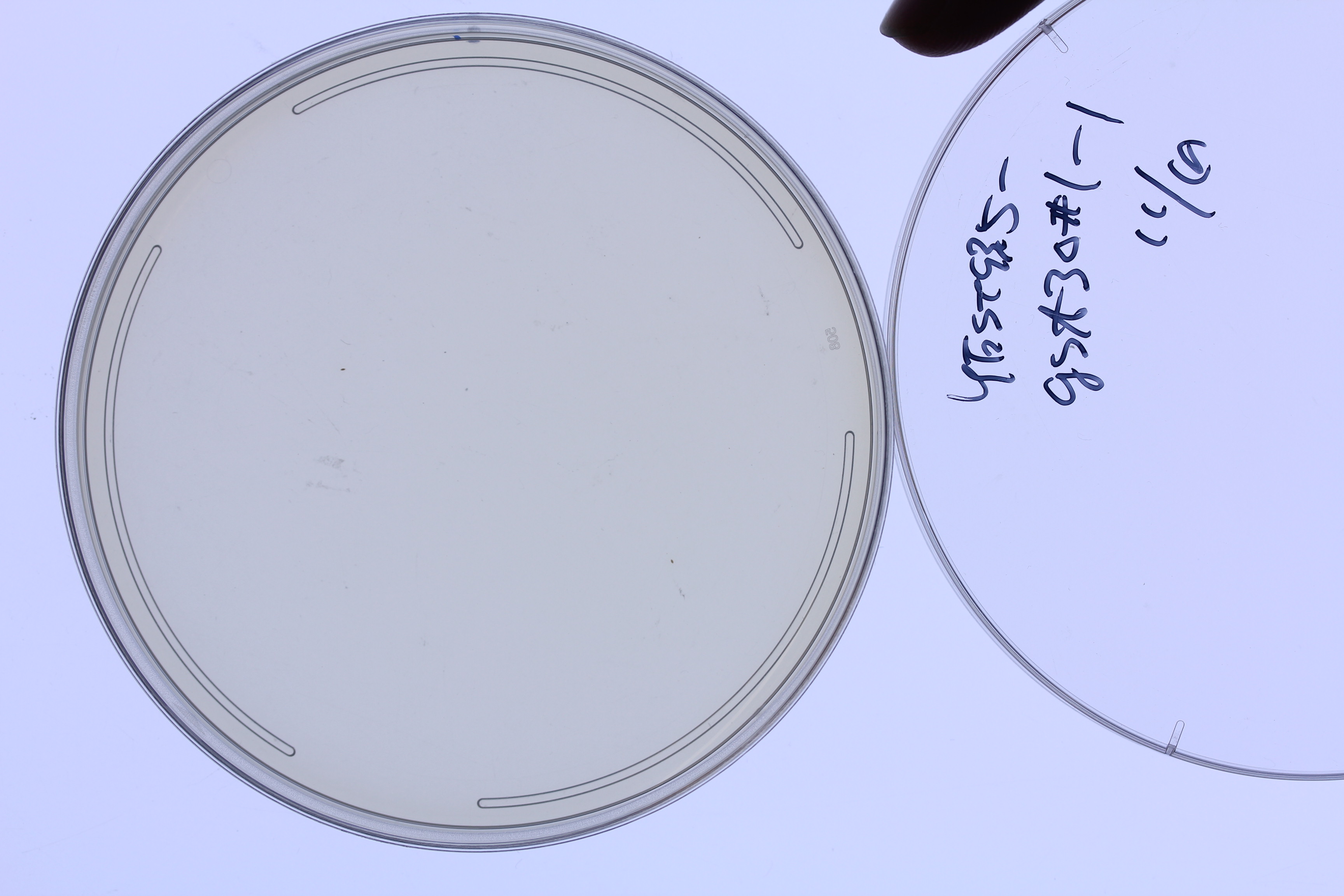

Supplement: Supplementary file 11 — Source data Fig. 5 [file 44318_2024_224_MOESM11_ESM.zip › EMBOJ-2024-117143-T-R_SourceData_Figure 5/ImageData/5J/YES93NH4Cl_gst3d_10day.tif]

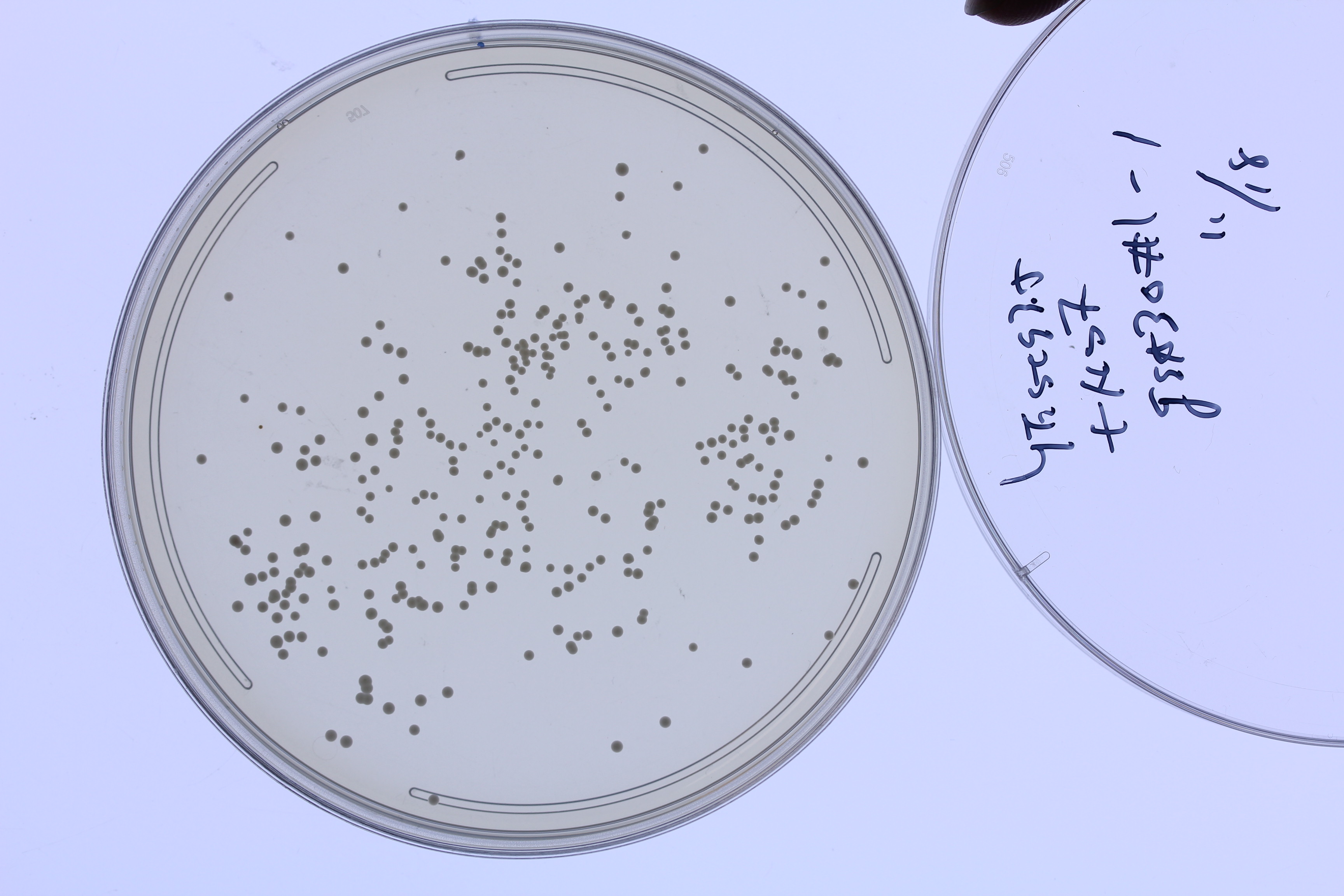

Supplement: Supplementary file 11 — Source data Fig. 5 [file 44318_2024_224_MOESM11_ESM.zip › EMBOJ-2024-117143-T-R_SourceData_Figure 5/ImageData/5J/YES93NH4Cl_NSF_gst3d_10day.tif]

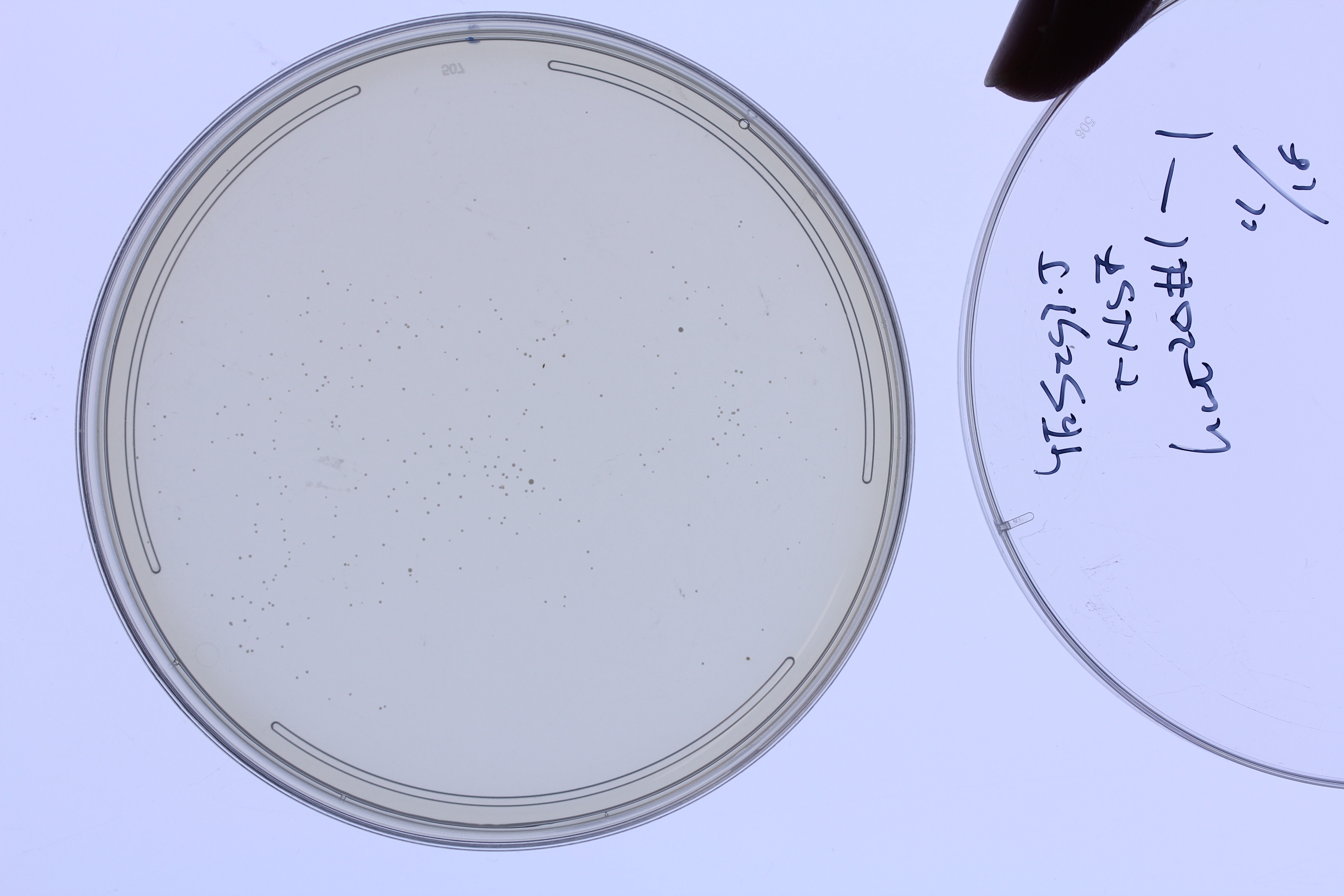

Supplement: Supplementary file 11 — Source data Fig. 5 [file 44318_2024_224_MOESM11_ESM.zip › EMBOJ-2024-117143-T-R_SourceData_Figure 5/ImageData/5J/YES93NH4Cl_NSF_hmt2d_10day.tif]

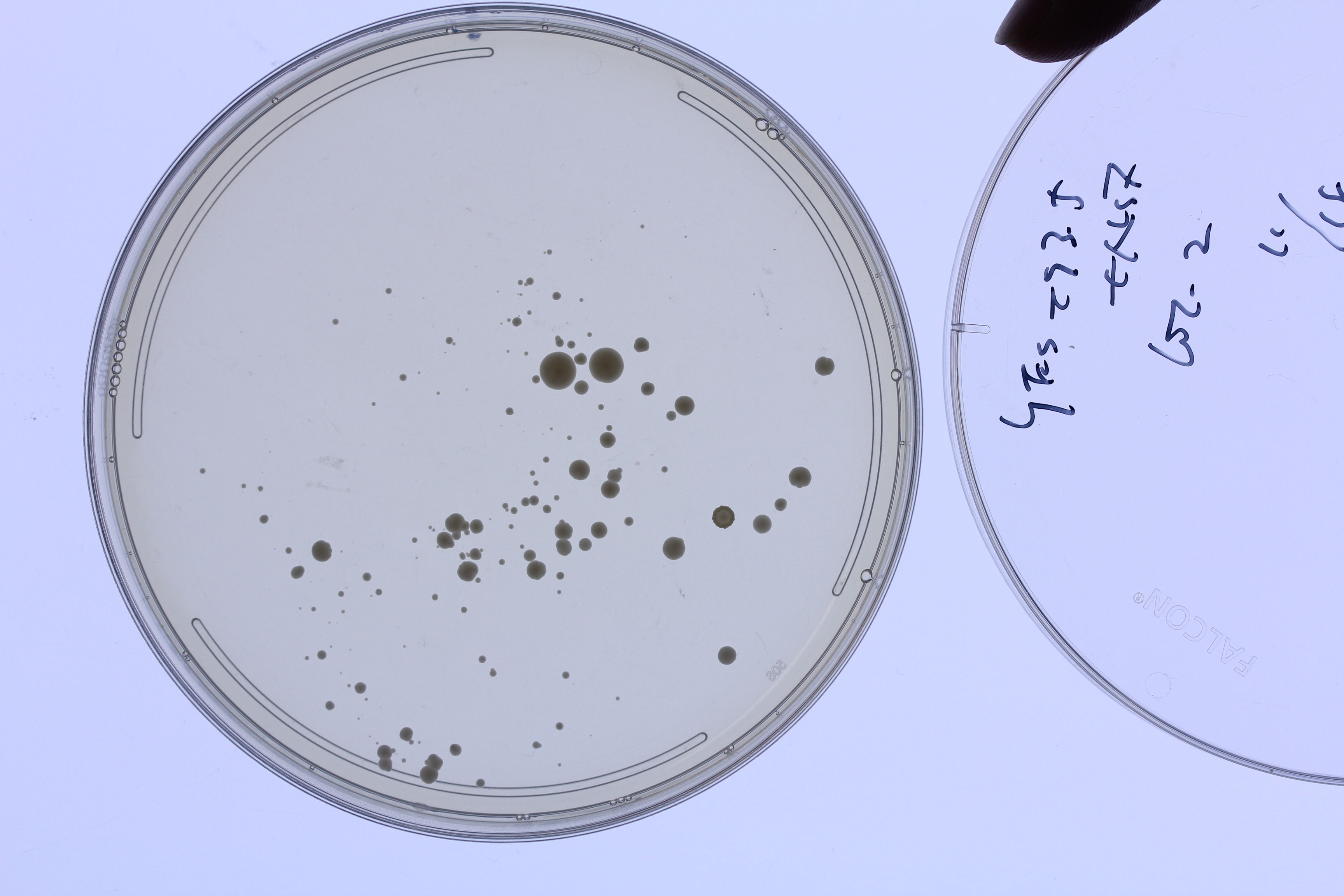

Supplement: Supplementary file 11 — Source data Fig. 5 [file 44318_2024_224_MOESM11_ESM.zip › EMBOJ-2024-117143-T-R_SourceData_Figure 5/ImageData/5J/YES93NH4Cl_NSF_WT_10day.tif]

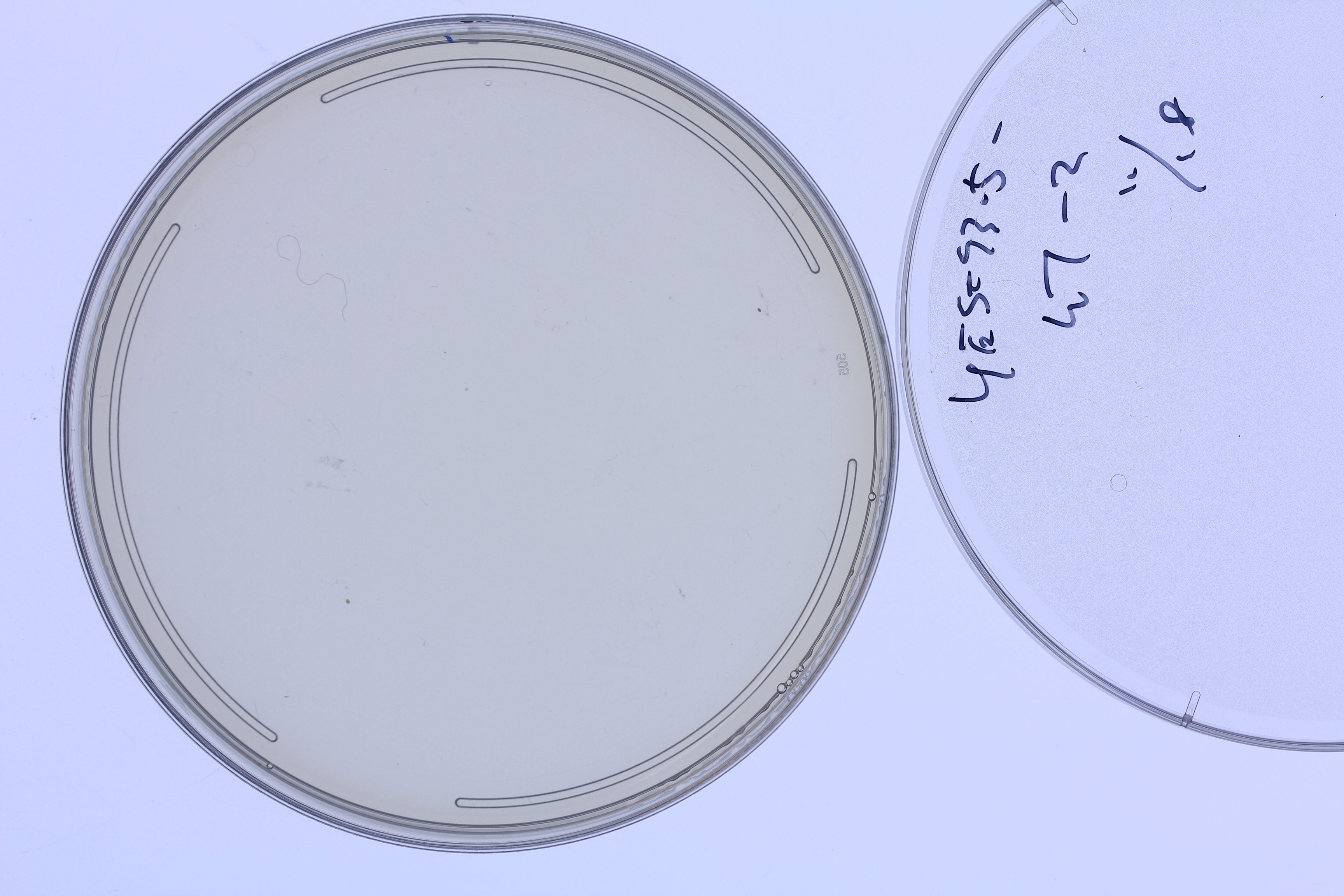

Supplement: Supplementary file 11 — Source data Fig. 5 [file 44318_2024_224_MOESM11_ESM.zip › EMBOJ-2024-117143-T-R_SourceData_Figure 5/ImageData/5J/YES93NH4Cl_WT_10day.tif]

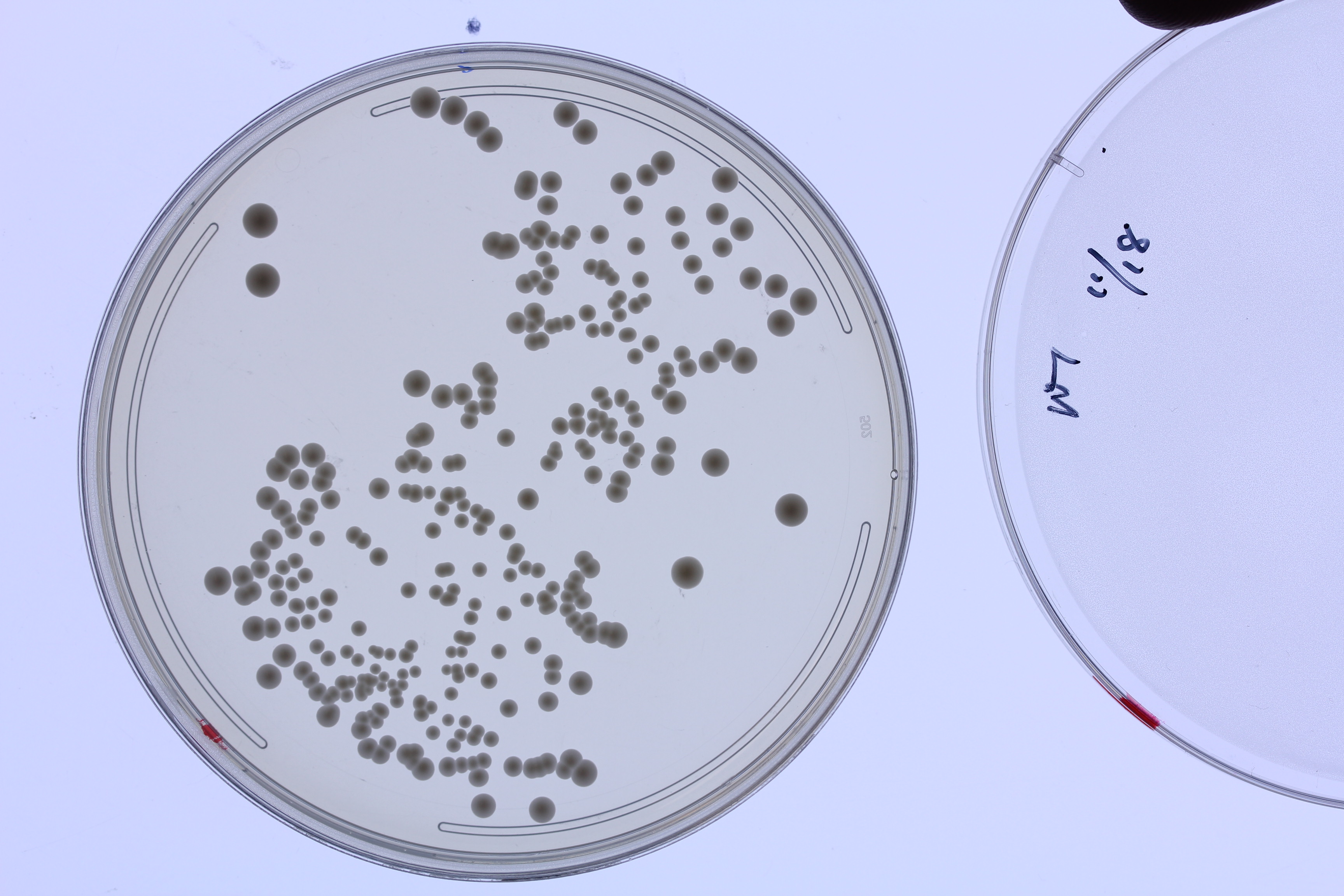

Supplement: Supplementary file 11 — Source data Fig. 5 [file 44318_2024_224_MOESM11_ESM.zip › EMBOJ-2024-117143-T-R_SourceData_Figure 5/ImageData/5J/YES_WT_5day.tif]

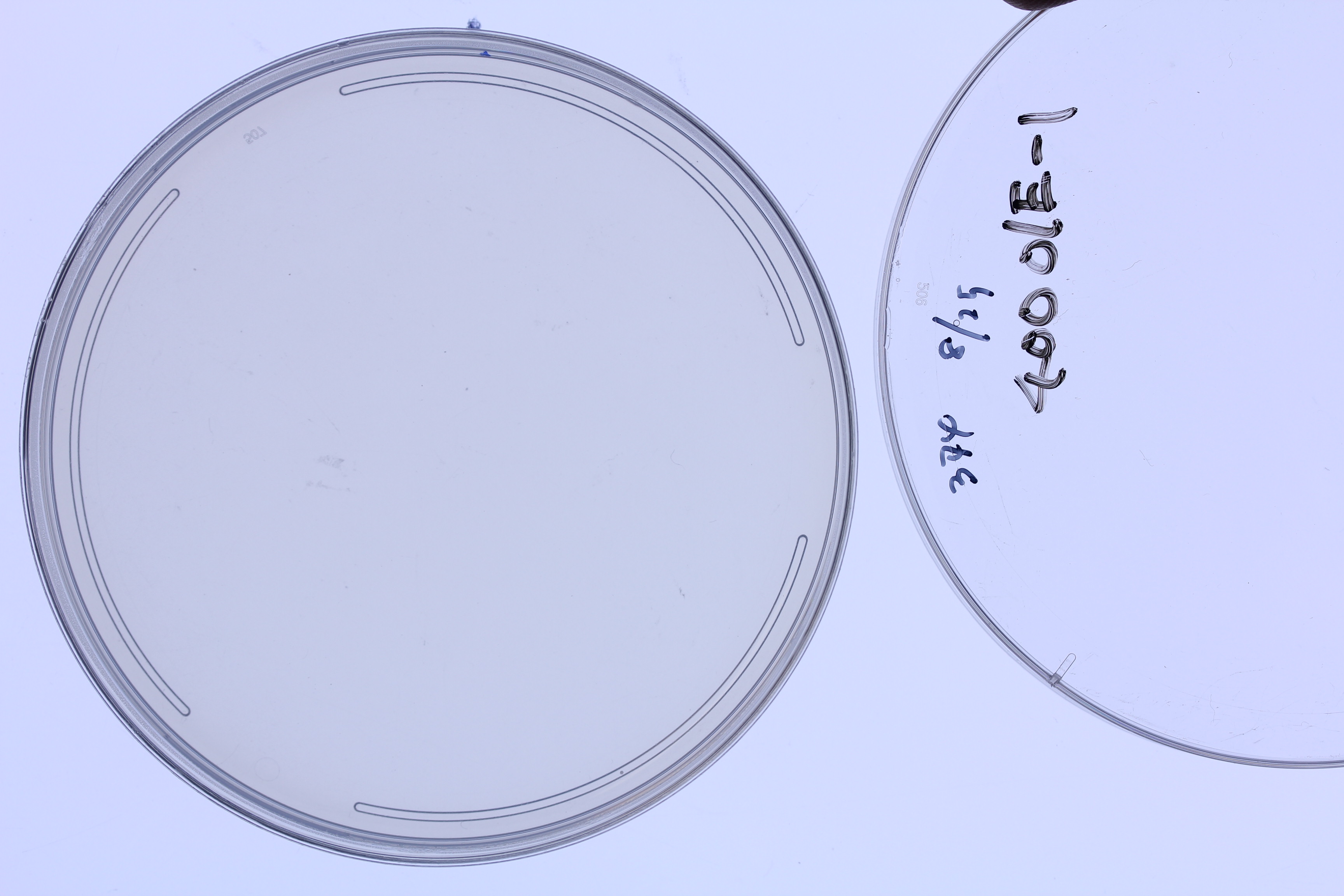

Supplement: Supplementary file 11 — Source data Fig. 5 [file 44318_2024_224_MOESM11_ESM.zip › EMBOJ-2024-117143-T-R_SourceData_Figure 5/ImageData/5B/374mMNH4Cl_400Ole_5day.tif]

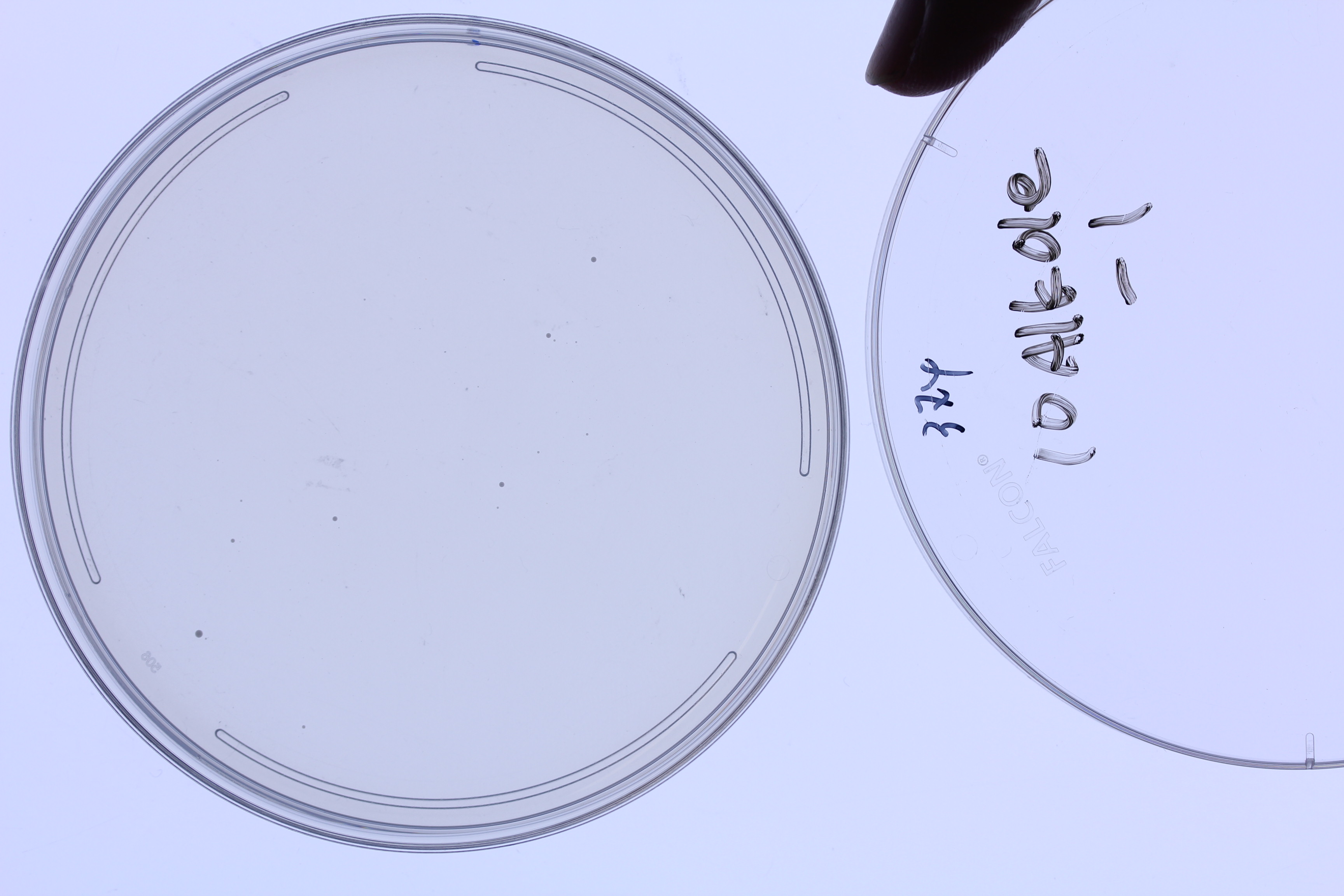

Supplement: Supplementary file 11 — Source data Fig. 5 [file 44318_2024_224_MOESM11_ESM.zip › EMBOJ-2024-117143-T-R_SourceData_Figure 5/ImageData/5B/374mMNH4Cl_10alkOle_5day.tif]

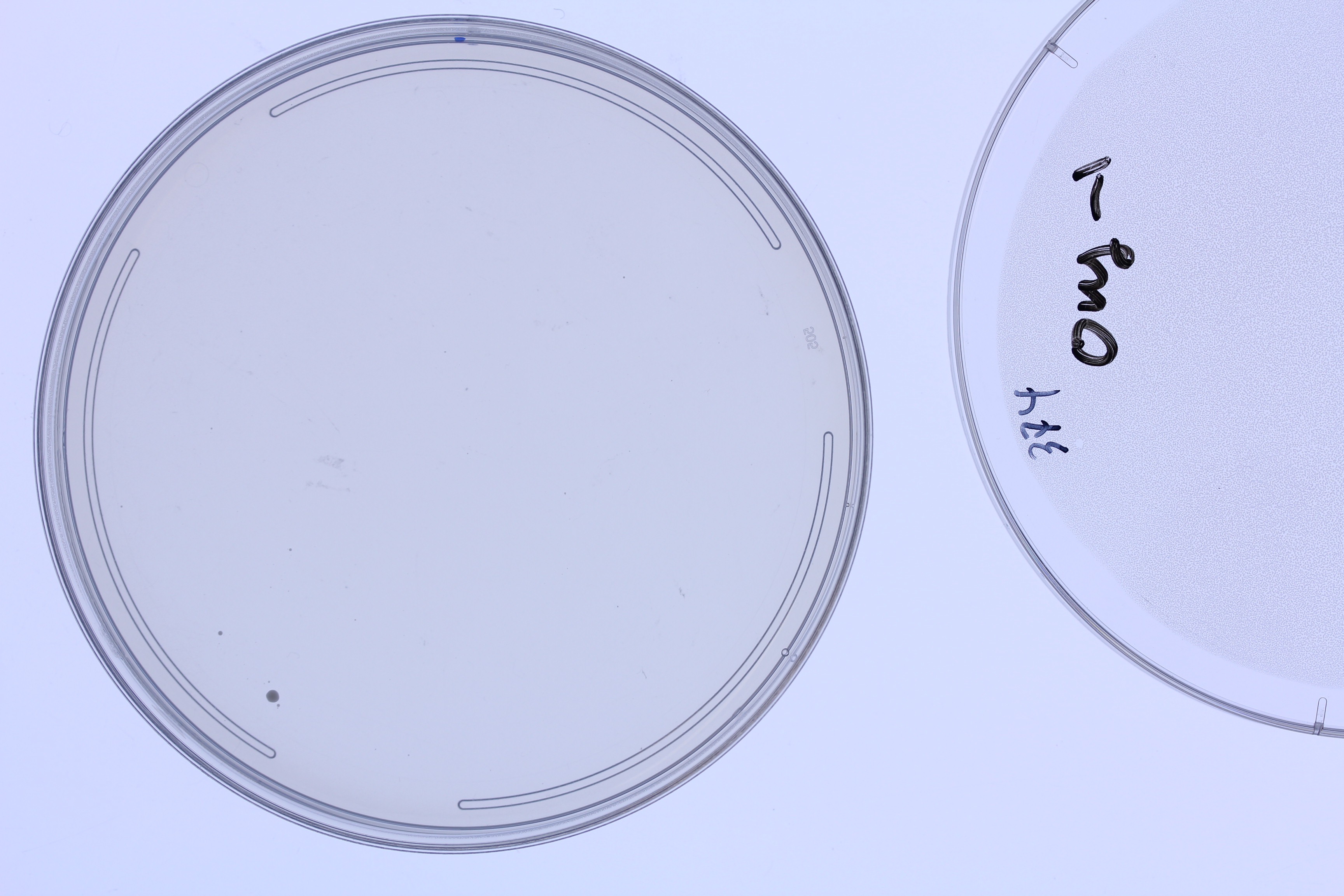

Supplement: Supplementary file 11 — Source data Fig. 5 [file 44318_2024_224_MOESM11_ESM.zip › EMBOJ-2024-117143-T-R_SourceData_Figure 5/ImageData/5B/374mMNH4Cl_5day.tif]

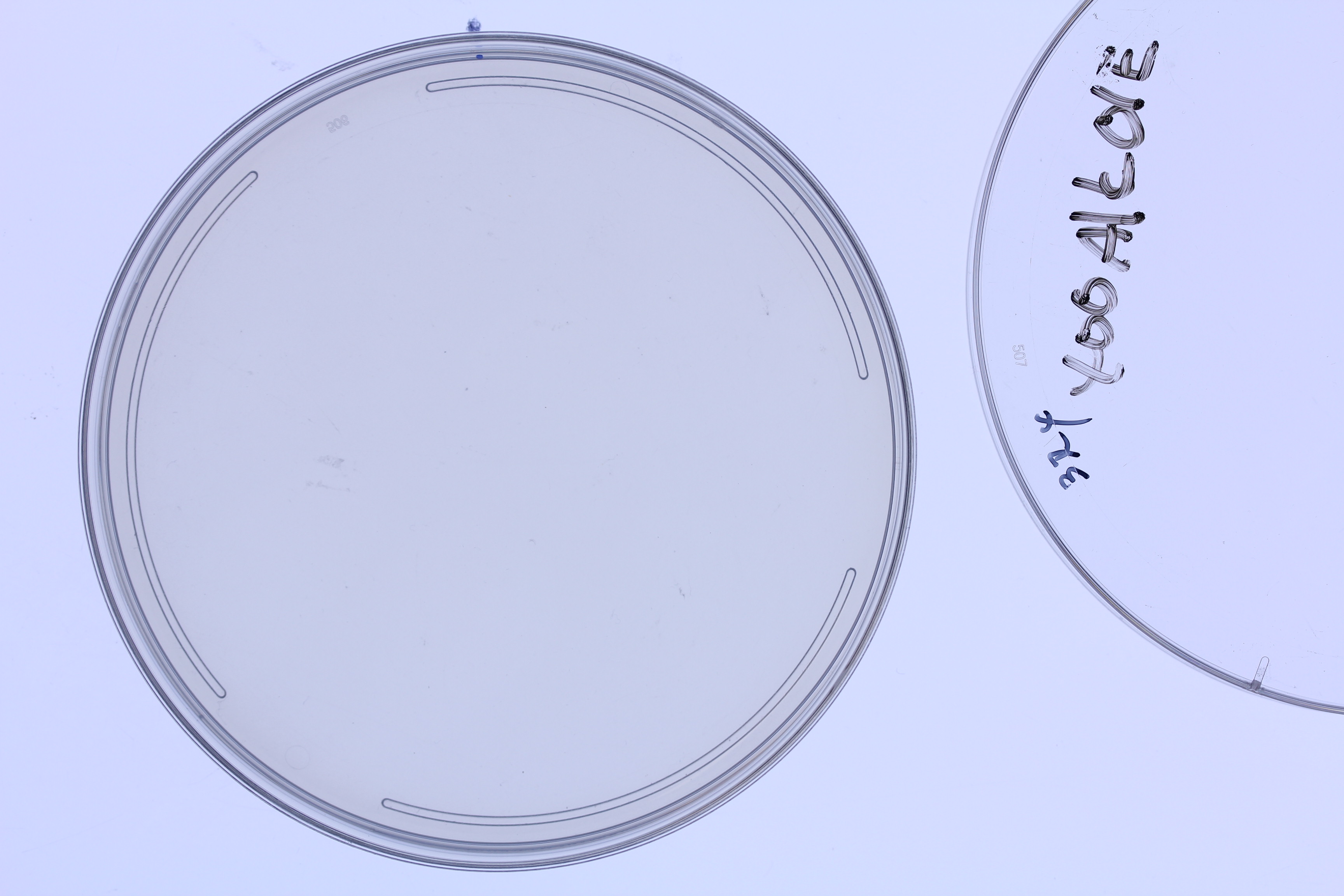

Supplement: Supplementary file 11 — Source data Fig. 5 [file 44318_2024_224_MOESM11_ESM.zip › EMBOJ-2024-117143-T-R_SourceData_Figure 5/ImageData/5B/374mMNH4Cl_400alkOle_5day.tif]

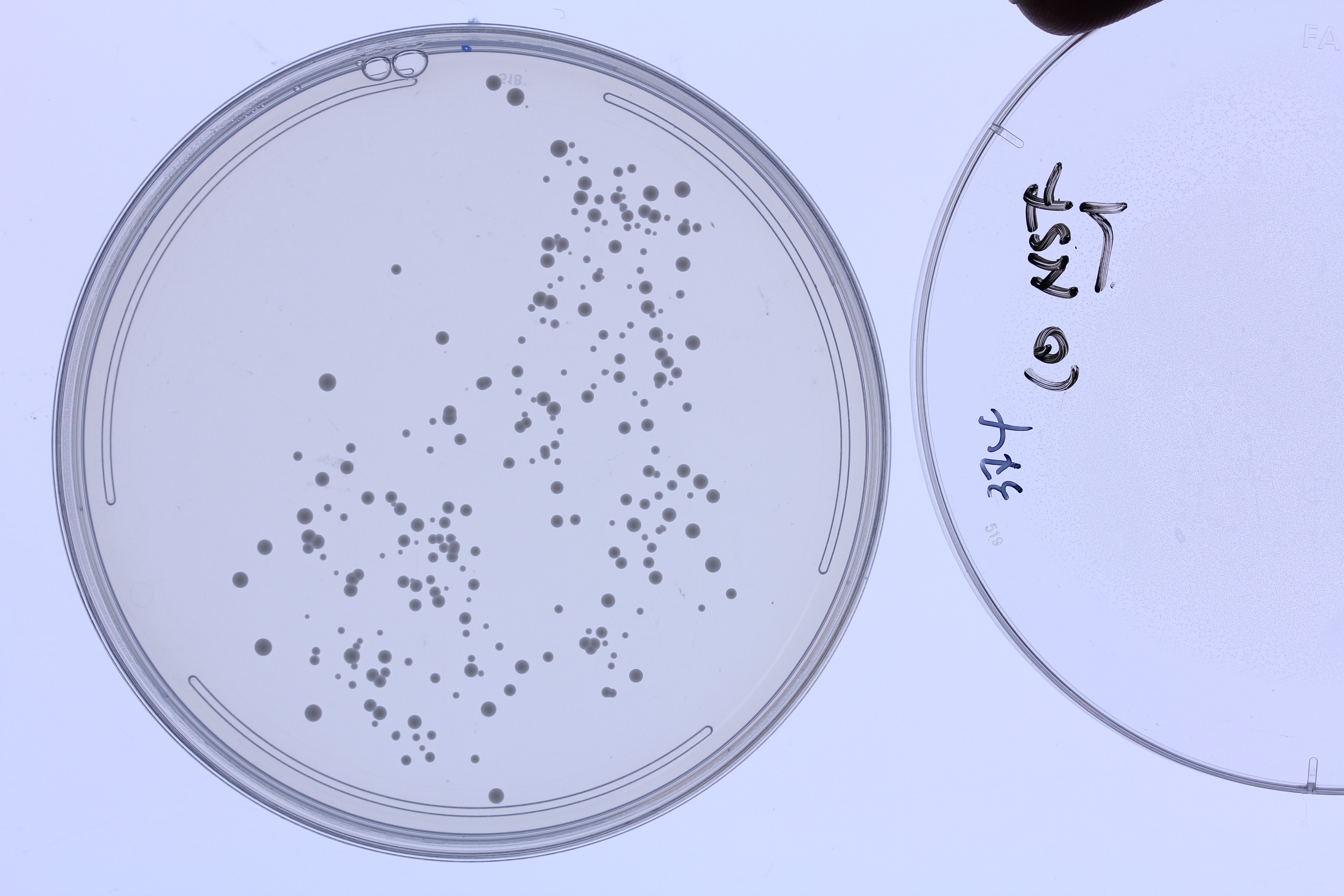

Supplement: Supplementary file 11 — Source data Fig. 5 [file 44318_2024_224_MOESM11_ESM.zip › EMBOJ-2024-117143-T-R_SourceData_Figure 5/ImageData/5B/374mMNH4Cl_10NSF_5day.tif]

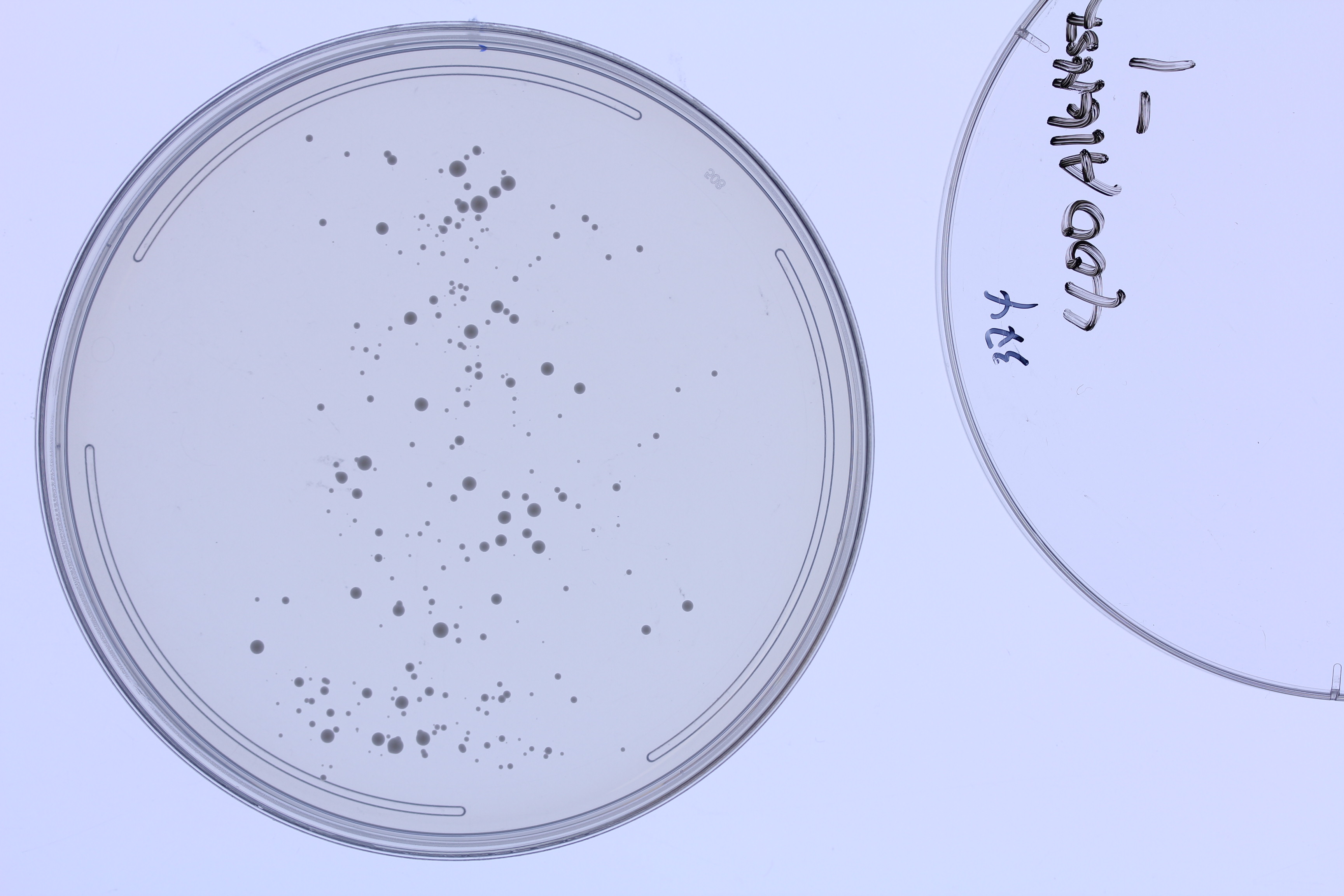

Supplement: Supplementary file 11 — Source data Fig. 5 [file 44318_2024_224_MOESM11_ESM.zip › EMBOJ-2024-117143-T-R_SourceData_Figure 5/ImageData/5B/374mMNH4Cl_400alkNSF_5day.tif]

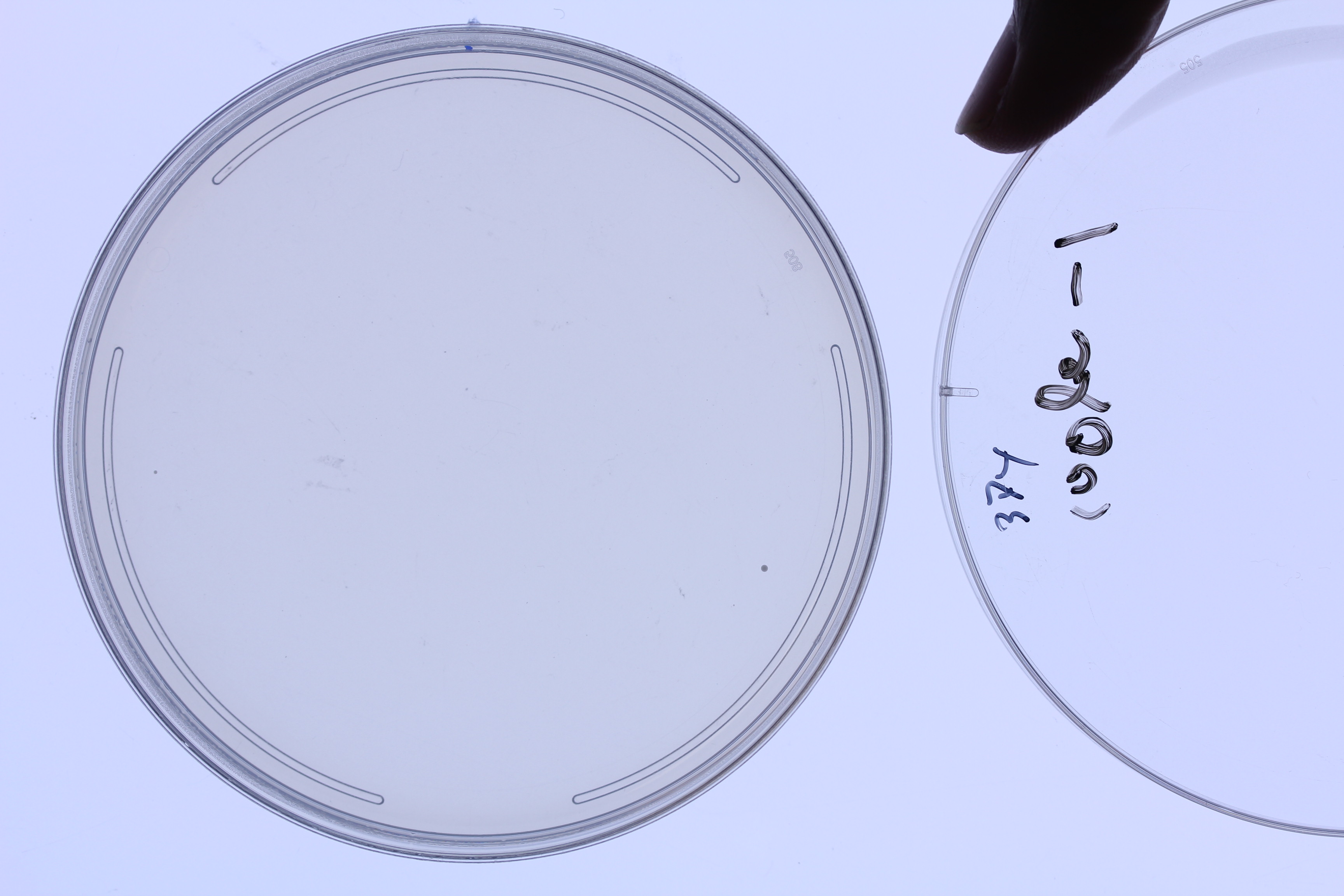

Supplement: Supplementary file 11 — Source data Fig. 5 [file 44318_2024_224_MOESM11_ESM.zip › EMBOJ-2024-117143-T-R_SourceData_Figure 5/ImageData/5B/374mMNH4Cl_10Ole_5day.tif]

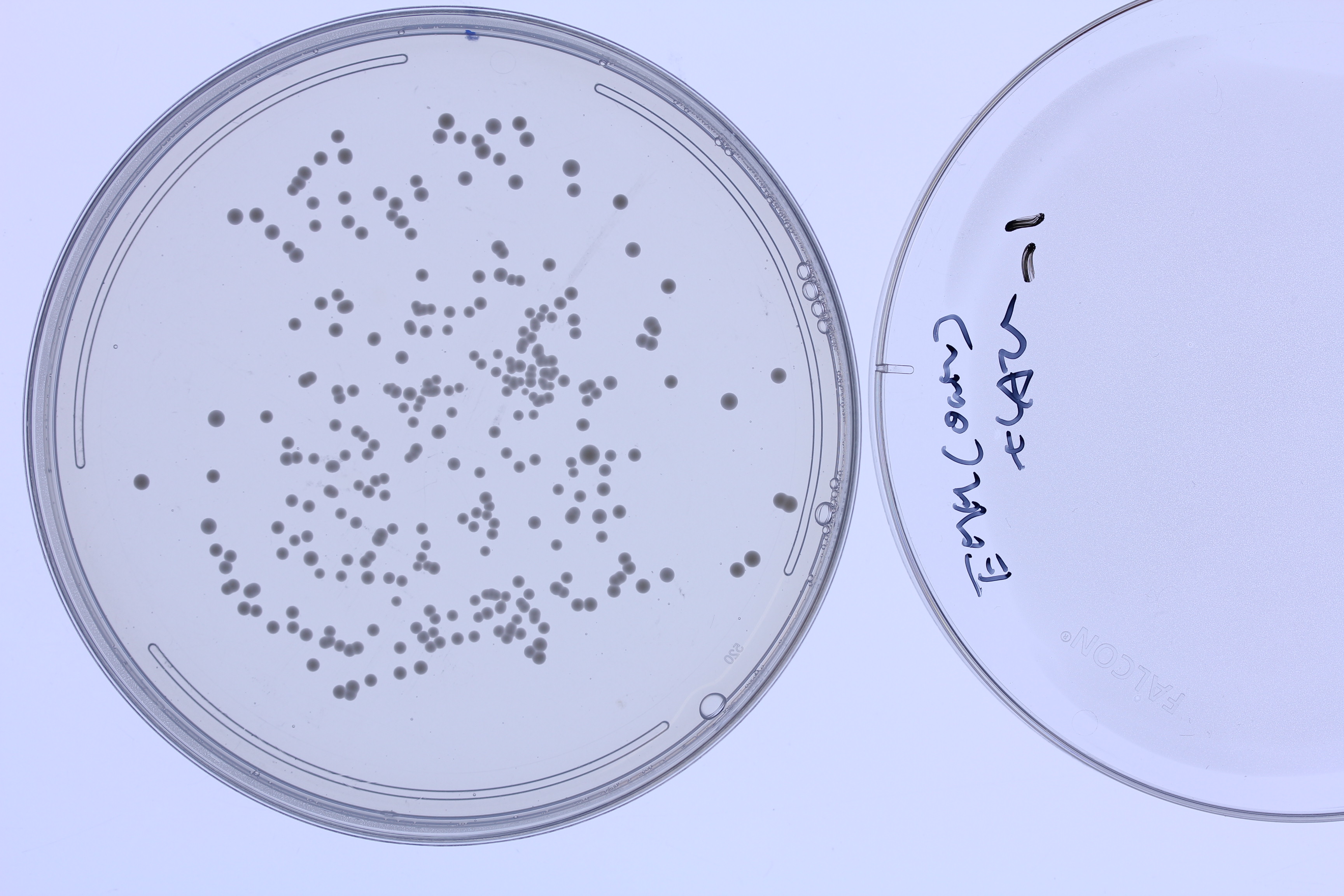

Supplement: Supplementary file 11 — Source data Fig. 5 [file 44318_2024_224_MOESM11_ESM.zip › EMBOJ-2024-117143-T-R_SourceData_Figure 5/ImageData/5B/0mMNH4Cl_5day.tif]

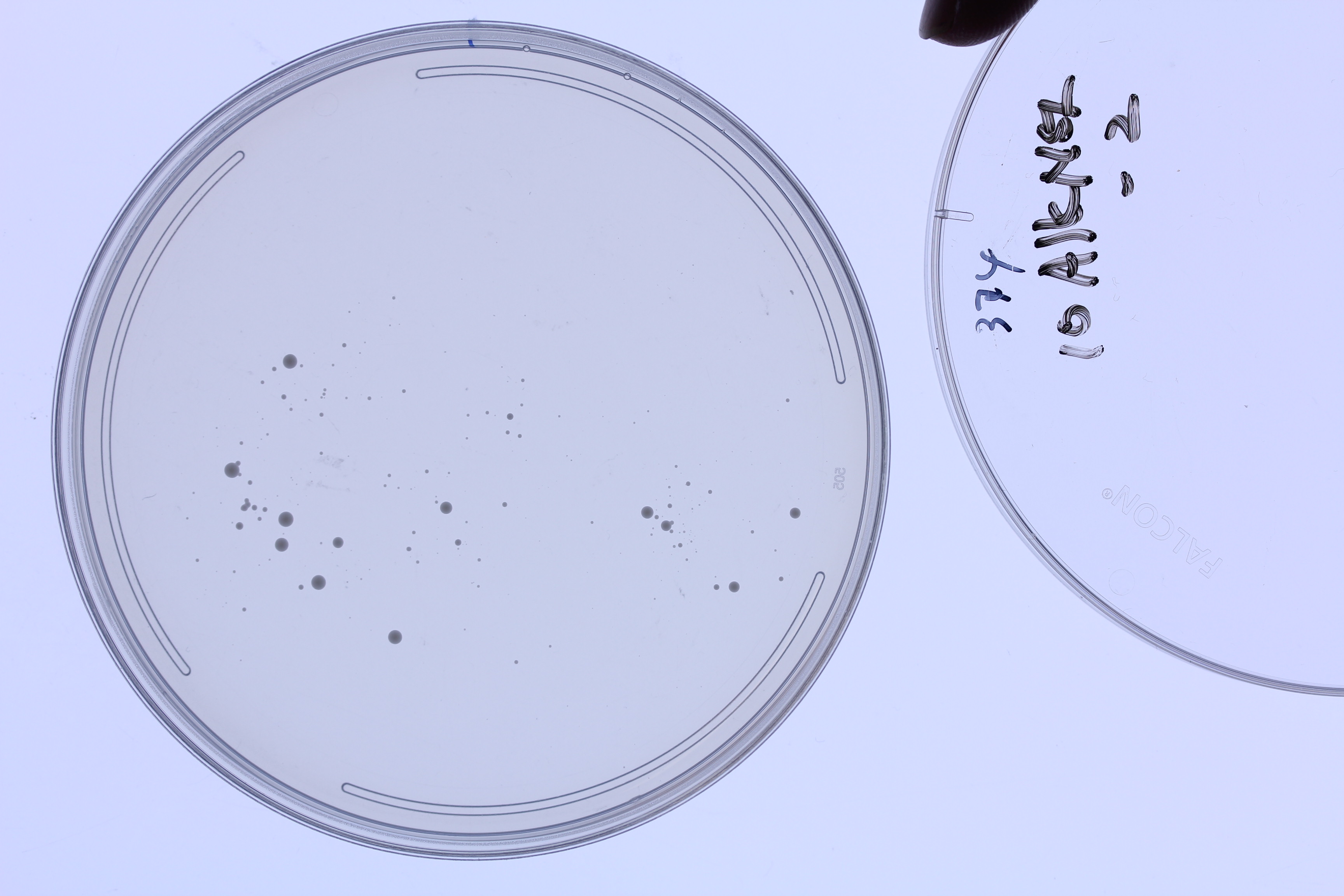

Supplement: Supplementary file 11 — Source data Fig. 5 [file 44318_2024_224_MOESM11_ESM.zip › EMBOJ-2024-117143-T-R_SourceData_Figure 5/ImageData/5B/374mMNH4Cl_10alkNSF_5day.tif]
