# Supplementary material for: Nitrogen signaling factor triggers a respiration-like gene expression program in fission yeast
Source: EMBO J. 2024 Sep 10;43(20):9. doi: 10.1038/s44318-024-00224-z (PMC11480445; doi:10.1038/s44318-024-00224-z)
Supplement: Supplementary file 12 — Expanded View Figures [file 44318_2024_224_MOESM12_ESM.pdf]

Expanded View Figures

Figure EV1. Differential gene expression analysis of cells treated with NSF for 4 or 6 h.

(**A**) Pairwise correlation plots comparing log2FC gene expression changes at different time points of NSF treatment. Correlation coefficients were calculated with Pearson's r. (**B**) MA plot showing differential gene expression (log2FC) in cells treated with MeOH or NSF for 4 or 6 h (y-axis). The 2-h treatment is shown in Fig. 2A. The x-axis denotes total transcript abundance in counts per million (cpm) in both conditions. *p* values were calculated using the Wald test and adjusted with the Benjamini and Hochberg method. Up- or down-regulated genes (FC >1.5 and adjusted *p* value <0.05) are highlighted in pink or blue, respectively. NSF-linked genes revealed by the genetic screen are marked with a black outline. The names of the top two upregulated and downregulated genes are written in red and blue, respectively. The green color was used to label the genes encoding transcription factors. (**C**) Heatmap of Pearson correlation coefficients between log2FC gene expression changes induced by NSF and glycerol feeding. Pearson's r values were calculated among log2FC gene expression changes of genes with a gene expression level higher than 100 cpm after a 2-h NSF treatment under NCR conditions ([NSF/MeOH]) from two repetitive experiments (Exp.1 and Exp.2) and glycerol feeding ([Gly/Glu]) (Malecki et al, 2016). (**D**) Box plots showing logFC distribution of NSF-induced gene expression changes upon 4 or 6 h NSF treatment, grouped by gene expression changes induced by respiration (Malecki et al, 2016). *p* values were calculated using a two-sided *t*-test and were adjusted using the Holm method. The center line inside the box represents the median of the data, bounds of the box correspond to the interquartile range with the bottom and top of the box indicating the first and third quartiles, and bounds of whiskers extend to the minimum and maximum values within the range of non-outlier data. Each condition was done in biological triplicates.

© The Author(s)

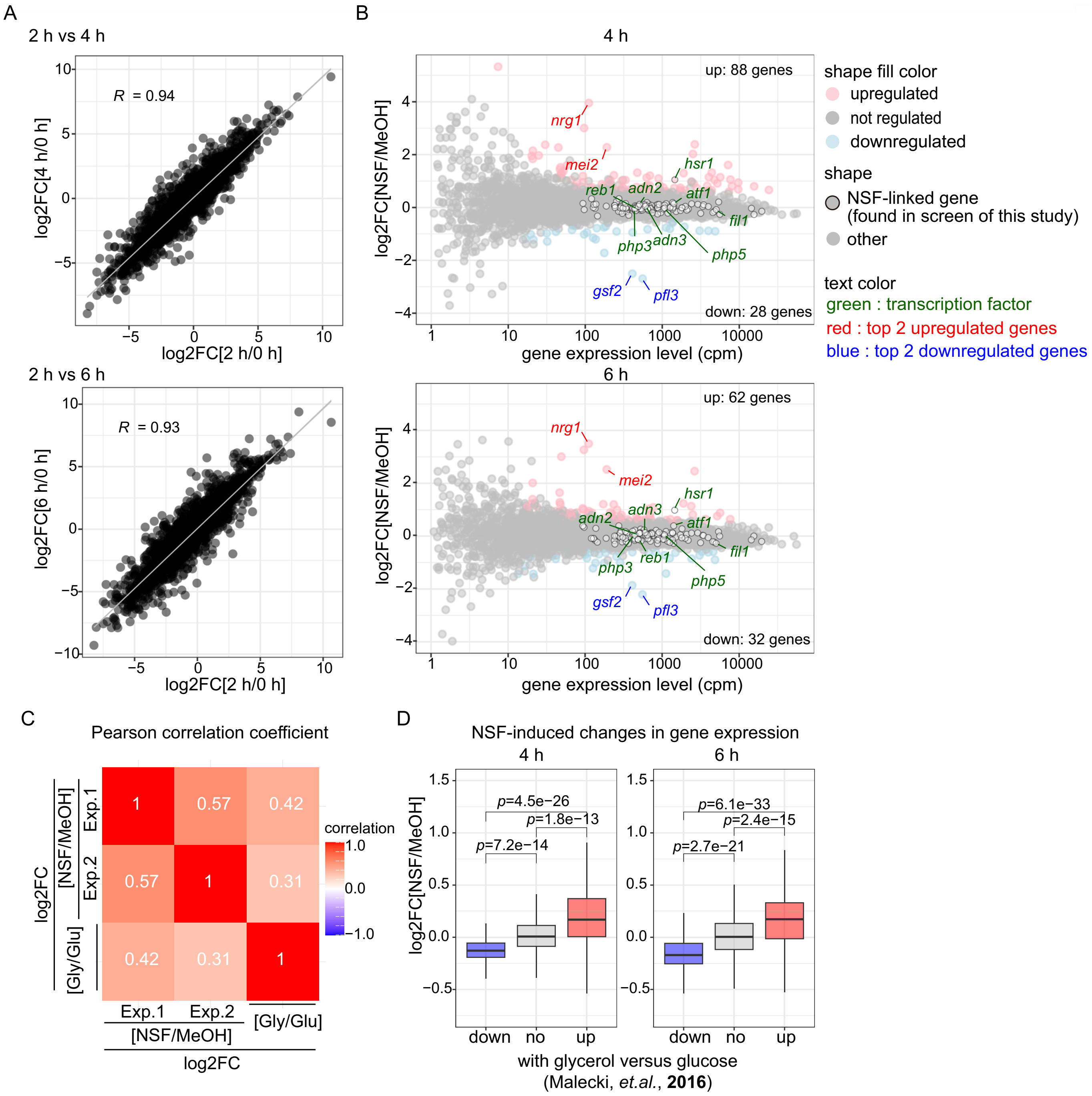

A
2 h vs 4 h
R = 0.94
log2FC[4 h/0 h]
log2FC[2 h/0 h]
2 h vs 6 h
R = 0.93
log2FC[6 h/0 h]
log2FC[2 h/0 h]
B
4 h
up: 88 genes
down: 28 genes
nrg1
mei2
hsr1
reb1
adn2
atf1
fil1
php3
adn3
php5
gsf2
pfl3
log2FC[NSF/MeOH]
gene expression level (cpm)
6 h
up: 62 genes
down: 32 genes
shape fill color
upregulated
not regulated
downregulated
shape
NSF-linked gene
(found in screen of this study)
other
text color
green : transcription factor
red : top 2 upregulated genes
blue : top 2 downregulated genes
C
Pearson correlation coefficient
correlation
log2FC
[NSF/MeOH]
Exp.1
Exp.2
[Gly/Glu]
1
0.57
0.42
0.31
D
NSF-induced changes in gene expression
4 h
6 h
p=4.5e−26
p=1.8e−13
p=7.2e−14
p=6.1e−33
p=2.4e−15
p=2.7e−21
log2FC[NSF/MeOH]
down
no
up
with glycerol versus glucose
(Malecki, et.al., 2016)

 © The Author(s)

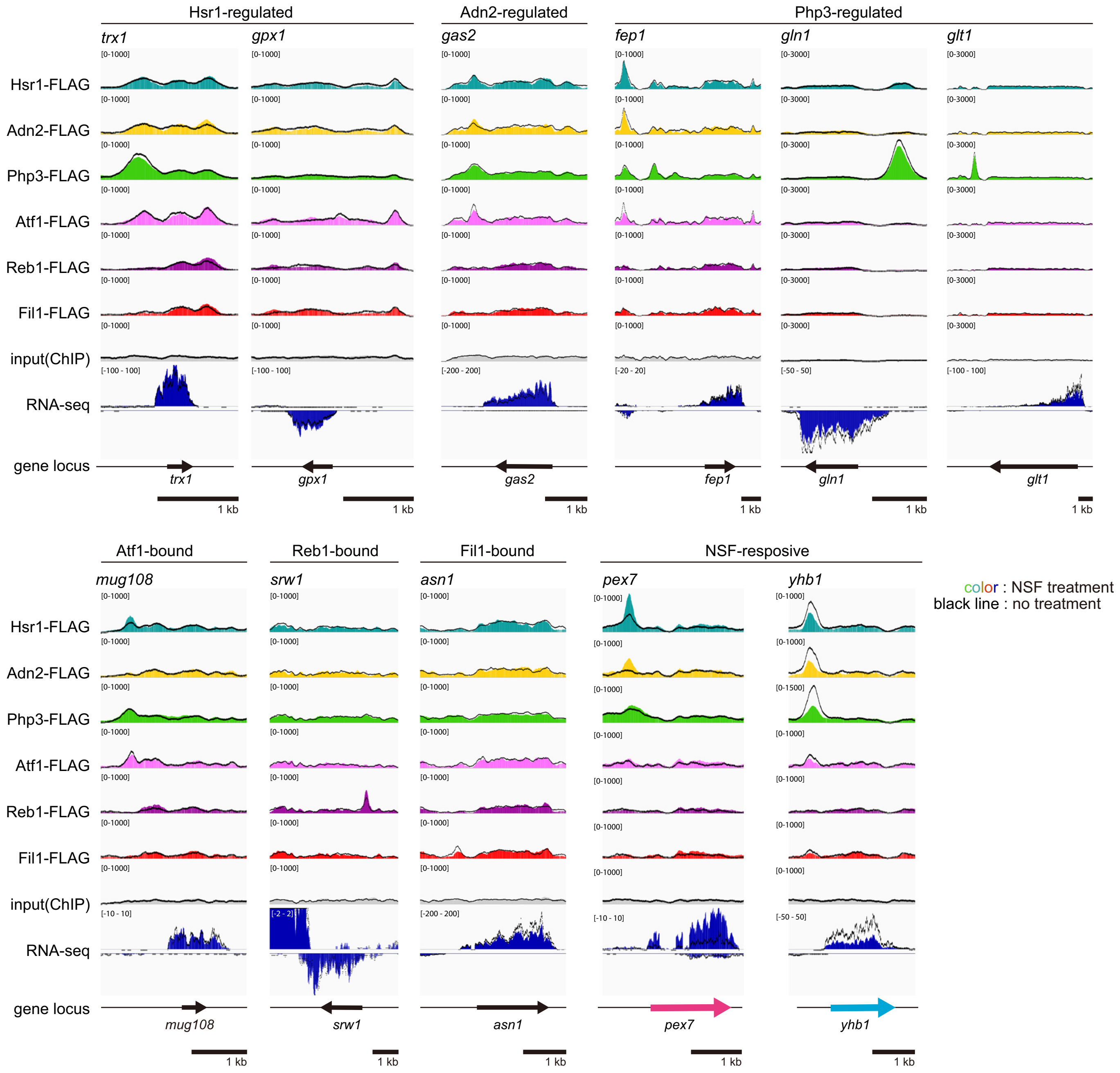

Figure EV2. ChIP-seq analysis of TFs required for NSF-mediated adaptive growth.

ChIP enrichments of Hsr1-FLAG, Adn2-FLAG, Php3-FLAG, Atf1-FLAG, Reb1-FLAG, and Fil1-FLAG on NSF-linked genes, showing occupancies of indicated TF on target or putative target genes (Hsr1: *trx1*, *gpx1* (Chen et al, 2008), Adn2: *gas2* (Kwon et al, 2012), Php3: *fep1, gln1, glt1* (Mercier et al, 2008, 2006), Atf1: *mug108* (Takemata et al, 2016), Reb1: *srw1* (Rodríguez-Sánchez et al, 2010), Fil1: *asn1* (Duncan et al, 2018)). Black lines denote ChIP enrichment in untreated cells. Colored areas represent ChIP enrichments upon NSF treatment. The RNA-seq tracks were derived from RNA-seq data of cells under NCR conditions with or without NSF for 2 h (see Fig. 2A).

© The Author(s)

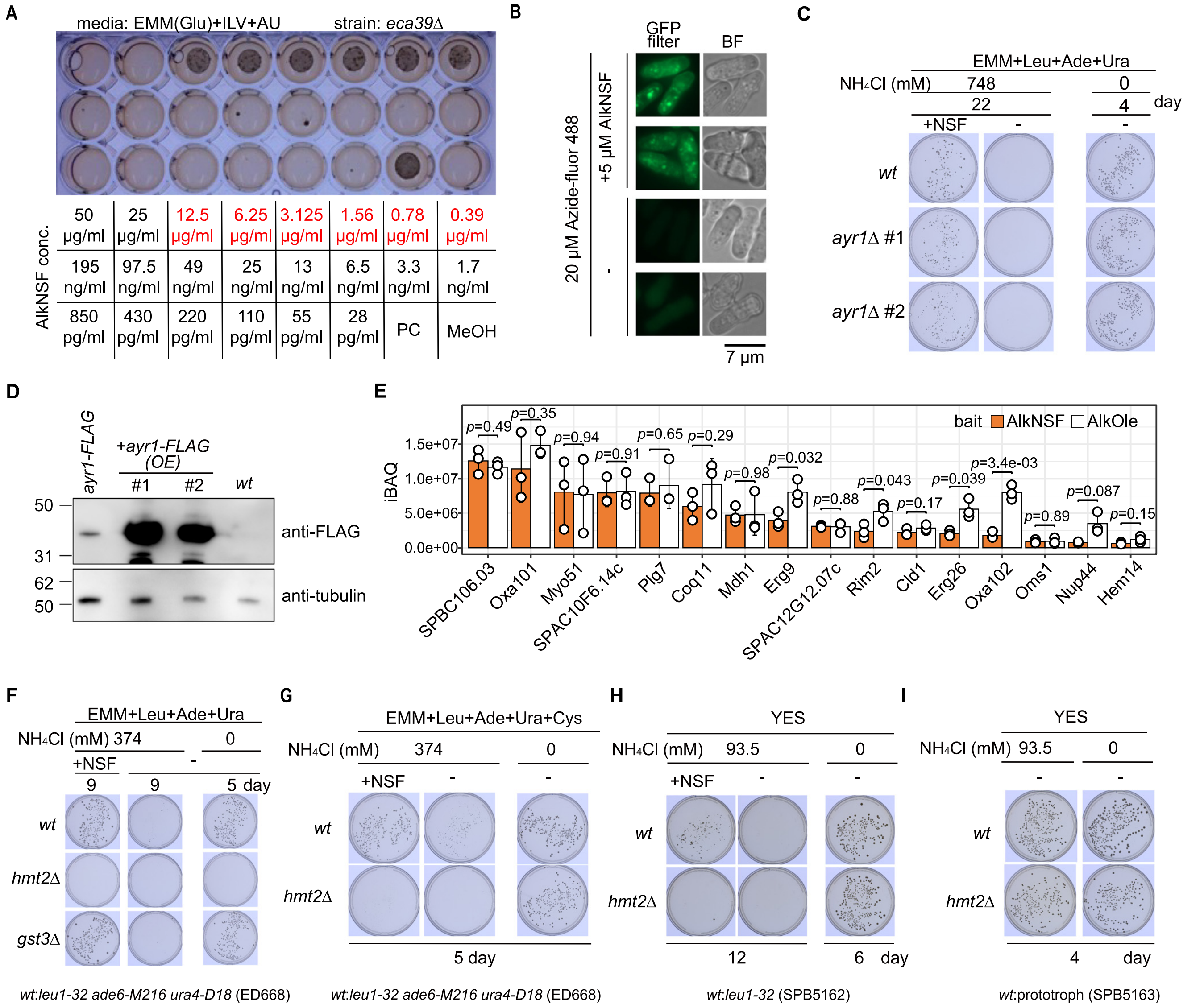

Figure EV3. Identification of NSF interacting proteins with a chemical biology probe.

(**A**) Determination of the minimum effective concentration of AlkNSF. *eca39Δ* cells were spotted onto EMM(Glu) + ILV + Ade + Ura with decreasing AlkNSF concentrations. As a positive control for this assay (PC), cells were exposed to secreted signaling factors that were purified from the supernatant of an *S. pombe* culture with the ethyl acetate method (Sun et al, 2016). 50% methanol (MeOH) served as a negative control. The plate was incubated for 6 days at 30 °C. (**B**) Visualization of cellular AlkNSF uptake by fluorescence microscopy. Cells were incubated with 5 μM AlkNSF in EMM(187 mM NH_4Cl) + Leu + Ade + Ura for 4 h. Subsequently, AlkNSF was conjugated with azide-flour 488 by click chemistry. (**C**) NSF-mediated adaptive growth of *wt* (ED668) or *ayr1Δ* (SPB5108 and SPB5109) cells in EMM medium. (**D**) Western blot analysis of Ayr1 protein levels in *wt* (ED668), *ayr1-FLAG* (SPB5122), and ayr1-FLAG overexpression (+*ayr1-FLAG (OE)*, SPB5227 and SPB5228) strains. Proteins were detected with anti-FLAG and anti-tubulin antibodies. (**E**) iBAQ values of proteins that co-purify with AlkNSF and AlkOle probes when incubated with wild-type cell lysates. The mean and standard deviation from technical triplicates is shown. *p* values were calculated using a two-sided *t*-test. (**F**) NSF-mediated adaptive growth of *wt* (ED668), *hmt2Δ* (SPB5150), or *gst3Δ* (SPB5158) cells in EMM medium. (**G**) NSF-mediated adaptive growth of *wt* (ED668) or *hmt2Δ* (SPB5150) cells in EMM medium supplemented with 2 mM cysteine. (**H**) NSF-mediated adaptive growth of *wt* (carrying only leucine auxotrophy: SPB5162), or *hmt2Δ* (SPB5234) cells in YES medium. (**I**) NSF-mediated adaptive growth of *wt* prototroph (SPB5163) or *hmt2Δ* (SPB5233) cells in YES medium.

 © The Author(s)

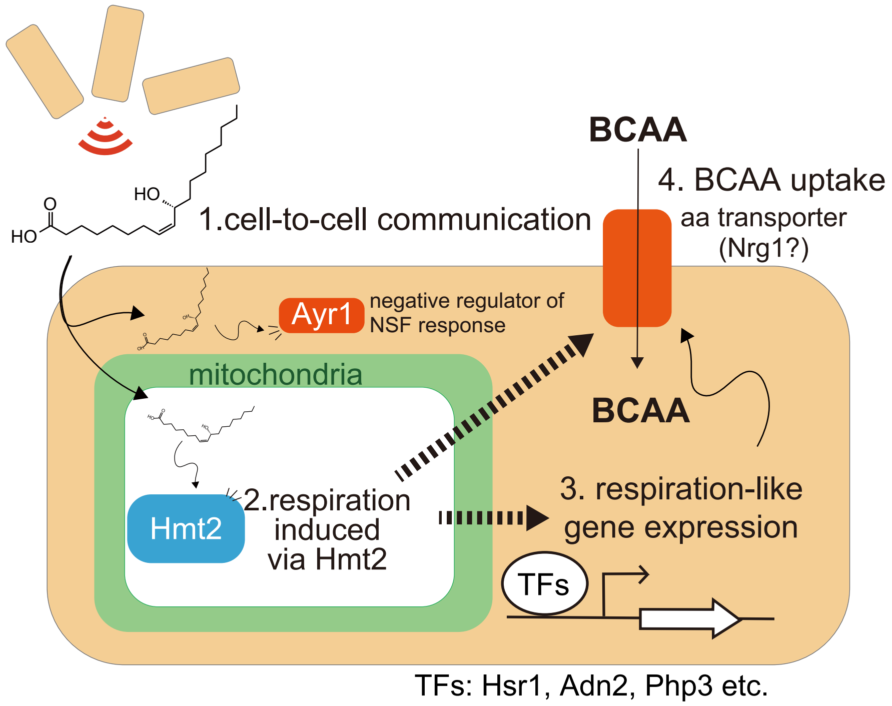

Figure EV4. Working model.

Schematic summary of the key findings of this study. NSF activates mitochondrial respiration by binding to Hmt2, eventually triggering changes in gene expression, the evasion of NCR, and the uptake of BCAA. Ayr1 regulates the response negatively, possibly by metabolizing NSF.
